# Dynamic miRNA changes during the process of epileptogenesis in an infantile and adult-onset model

**DOI:** 10.1038/s41598-021-89084-9

**Published:** 2021-05-06

**Authors:** Petra Bencurova, Jiri Baloun, Jakub Hynst, Jan Oppelt, Hana Kubova, Sarka Pospisilova, Milan Brazdil

**Affiliations:** 1 CEITEC - Central European Institute of Technology, Masaryk University, Kamenice 5, 625 00 Brno, Czech Republic; 2 Brno Epilepsy Center, Department of Neurology, St. Anne’s University Hospital and Medical Faculty of Masaryk University, Pekarska 53, 656 91 Brno, Czech Republic; 3 Department of Developmental Epileptology, Institute of Physiology, Academy of Sciences Czech Republic, Videnska 1083, 14220 Prague, Czech Republic; 4 Department of Pathology and Laboratory Medicine, Division of Neuropathology, Perelman School of Medicine, University of Pennsylvania, 19104-6100 Philadelphia, PA USA

**Keywords:** Neuroscience, Diseases of the nervous system, Molecular neuroscience

## Abstract

Temporal lobe epilepsy (TLE) is the most common epilepsy type. TLE onset in infancy aggravates features like severity, drug responsiveness, or development of comorbidities. These aggravations may arise from altered micro RNA (miRNA) expression specific to the early onset of the disease. Although the miRNA involvement in TLE is widely studied, the relationship between the onset-age and miRNA expression has not been addressed. Here, we investigated the miRNA profile of infantile and adult-onset TLE in rats combining sequencing and PCR. Since miRNA expression changes with the disease progression, we scrutinized miRNA dynamics across three stages: acute, latent, and chronic. We report that infantile-onset TLE leads to changes in the expression of fewer miRNAs across these stages. Interestingly, the miRNA profile in the acute stage of infantile-onset TLE overlaps in dysregulation of miR-132-5p, -205, and -211-3p with the chronic stage of the disease starting in adulthood. The analysis of putative targets linked the majority of dysregulated miRNAs with pathways involved in epilepsy. Our profiling uncovered miRNA expression characteristic for infantile and adulthood-onset epileptogenesis, suggesting the distinct biology underlying TLE in the onset age-dependent matter. Our results indicate the necessity of addressing the onset age as an important parameter in future epilepsy research.

## Introduction

Temporal lobe epilepsy (TLE) often originates in early childhood or infancy when the brain is undergoing rapid development. The early onset of TLE escalates the risk of medical intractability and induces a variety of epileptic comorbidities^1,2^. These features may arise from deviations in gene expression regulation accompanying the onset and progression of epilepsy. Hence, disease onset in the immature brain can aggravate brain development via altered expression of genes.

Defects in gene expression regulation are often caused by an imbalance in microRNA (miRNA) levels. miRNAs belong to the family of short non-coding RNAs responsible for post-transcriptional regulation of gene expression^3^ and their fine-tuned activity is essential for brain development and function^4^. miRNA expression is altered in many neuropathologies including TLE^5^. Thus, miRNA therapy is widely studied as a potential alternative to current epilepsy treatment with promising results in rodent models^6^.

Although children and adults share many similar symptoms of temporal seizures, there are also differences arising from unique characteristics of the developing brain. The majority of studies address the miRNA expression, function, and treatment potential in epilepsy-induced only in adult animals without considering disease onset in childhood and infancy^5,6^. Therefore, these studies may neglect aspects essential for understanding epileptogenesis in the developing brain and undermine the risks and effectiveness of treatment.

In the present study, we have scrutinized miRNA profiles in three stages of epileptogenesis (acute, latent, and chronic) in order to identify similarities and differences between TLE with onset in developing and adult rat brain. Consistent with our previous study, we have analysed mature miRNA expression of the entire hippocampus (epileptic onset zone in TLE) combining two independent methods—Massive Parallel Sequencing (MPS) and qPCR in order to enhance the precision, specificity, and accuracy of miRNA profiling^7^. The comparison of miRNA expression throughout epileptogenesis in the developing and the mature brain sheds unique insight on epilepsy-induced alterations of gene expression regulation, suggesting a possible explanation for the differences arising from the age of disease onset.

## Results

In total, 83 animals aged P60 (32 controls, 51 pilocarpine-treated) were used for this study. 35 animals among those injected with pilocarpine developed motor status epilepticus (SE, mean latency 1045 ± 89 s). Two animals died after SE. We detected at least one motor seizure Racine stage 3–5 in 11 out of 13 SE animals during one week of continuous video-monitoring in the chronic epilepsy stage (Fig. S1). On average, monitored animals exhibited 5.1 seizures per week (min = 1, max = 23). In addition to Racine stage 3 seizures, one animal exhibited three generalized tonic–clonic seizures, one did not exhibit any motor seizures and one animal was euthanized because of a tumour during the monitoring period. These animals were excluded from analyses. Animals included in the control group did not exhibit motor seizures.

We used 30 control and 32 pilocarpine-treated animals in the P12 group. All animals receiving pilocarpine developed motor SE, while two of them died within one week after SE induction. Latency to motor SE was 635 ± 36 s (mean ± SE). In line with our previous studies, animals with SE at P12 did not develop motor seizures. No outliers in seizure occurrence were detected in P12 group that would require exclusion from the study. In P10-P12 rats, LiCl/pilocarpine SE results in the development of non-convulsive, electrographic seizures later in life. Using video/EEG monitoring for one week, we previously detected seizures in up to 87% of animals seven months after SE^8,9^.

### Whole miRNome profiling

**Figure 1:**
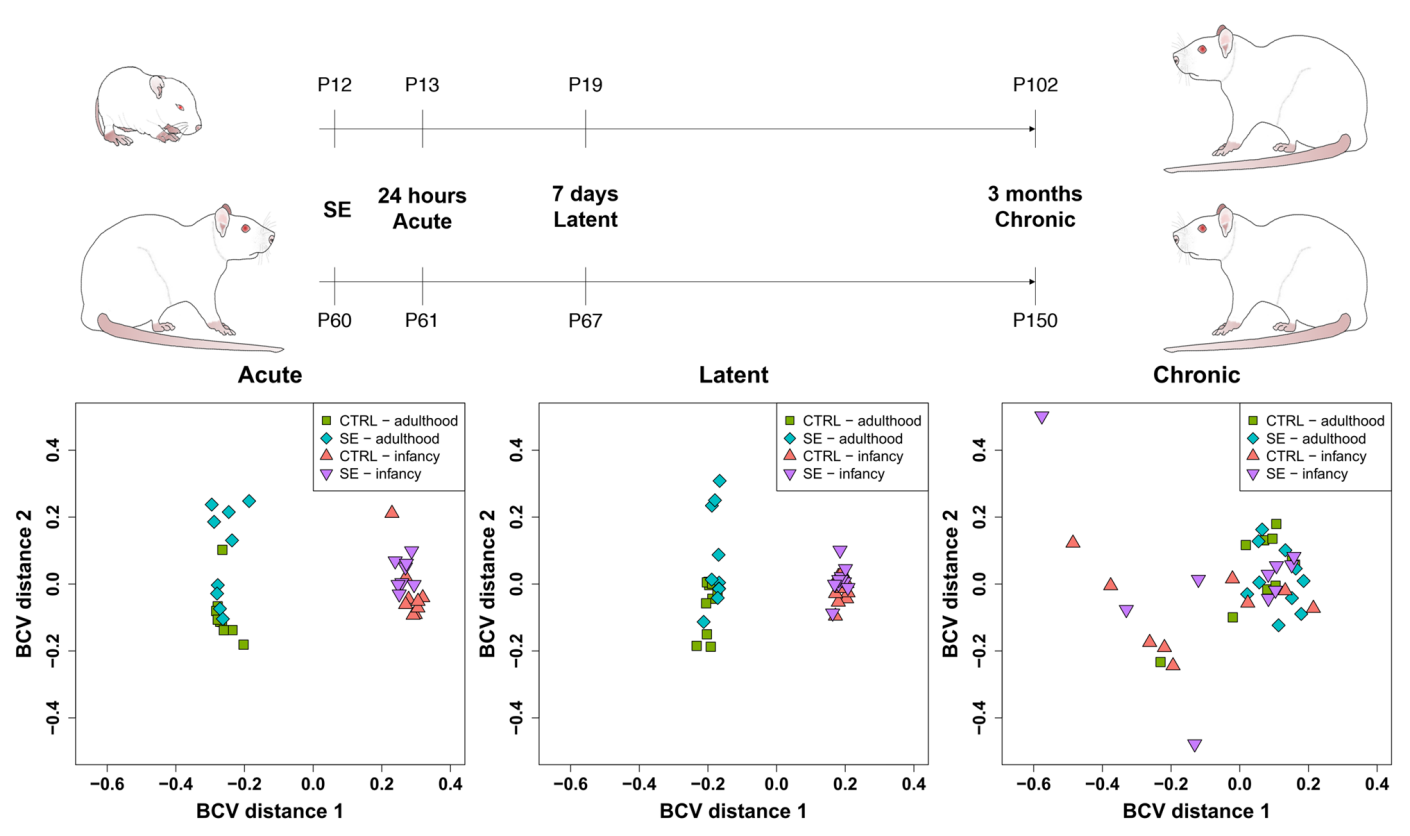
Timelines and Multidimensional Scaling (MDS) of complete miRNA expression. Timelines depict the age of animals (as a number of postnatal days P) at the status epilepticus (SE) induction and tissue collection separately for individual onset ages (infancy and adulthood). Tissue collection timepoints (24 h, 7 days, and 3 months) correspond with epileptogenesis stages: acute, latent, and chronic. The MDS plot is a clustering method to compute the relative variability of expression among samples and visualize the level of their similarity. The MDS plots were constructed using miRNA expression data (normalized read count from sequencing) and used “Biological coefficient of variation” (BCV) to compute distances. Points represent samples of the adult-onset (blue) and infantile-onset (violet) onset of epilepsy and their controls (adult—green, infantile—red). Individual plots depict clustering in a specific stage of epileptogenesis.

We used massive parallel sequencing (MPS) to scrutinize miRNA expression in the hippocampus of rats (n = 120) with epilepsy onset in infancy (P12) and adulthood at three different stages of epileptogenesis: (a) acute; (b) latent; and (c) chronic. Samples had median raw read coverage of 5.4 million, which dropped to 2.6 million clean potential miRNA reads (~ 53.91% of the raw reads) after adapter trimming, size selection, and removal of possible contaminants. Mapping to miRBase (v22) revealed the median content of miRNAs in the samples of 2.3 million (50.45% of the raw reads). Samples with less than 300 000 mapped miRNA reads (n = 6) were excluded from the differential expression analysis, while each group retained 8–10 samples. The average Phred quality score of all the samples was above 35 signifying very high sequencing quality. The total sum of ambiguous base content was < 0.01%. Sequencing identified 666 unique miRNA species across all samples. The number of miRNAs with raw read count over 500 gradually raised from the acute to the chronic stage (ranging from 331 to 419) in both age groups (Table 1). Moreover, multidimensional scaling (MDS) indicated a unique miRNA expression of the mature and developing brain. Samples belonging to the adult-onset and infantile-onset groups formed two separate clusters in early time-points (acute and latent stage) while merging in the 3-month time point (chronic stage), in which brains of rats in both age groups were fully developed (Fig. 1).

**Table 1:** Counts of miRNAs identified *DESEq2* and *limma*.

Epilepsy onset	Epilepsy stage	Raw reads > 500		Normalised reads > 500		*p*-value < 0.05 FC > 1.4 reads > 500		*p*-value < 0.01 FC > 1.4 reads > 500	
		DESeq2	limma	DESeq2	limma	DESeq2	limma	DESeq2	limma
Adulthood	Acute	334	348	313	335	36	40	29	40
	Latent	370	380	359	371	42	36	37	36
	Chronic	394	400	380	385	38	30	21	30
Infancy	Acute	331	339	324	311	27	25	21	25
	Latent	365	380	368	378	3	3	1	3
	Chronic	407	419	376	395	12	15	4	15

Descriptive results of two workflows of massive parallel sequencing data processing: *DESeq2* and *limma*. The table contains count of miRNAs reaching threshold of 500 reads (both raw and normalised) in each statistical analysis. Counts of miRNAs with fold-change over 1.4, *p*-value below 0.05 or 0.01 and number of normalised reads above 500 are displayed for each stage of epileptogenesis in both age groups respectively.

**Figure 2:**
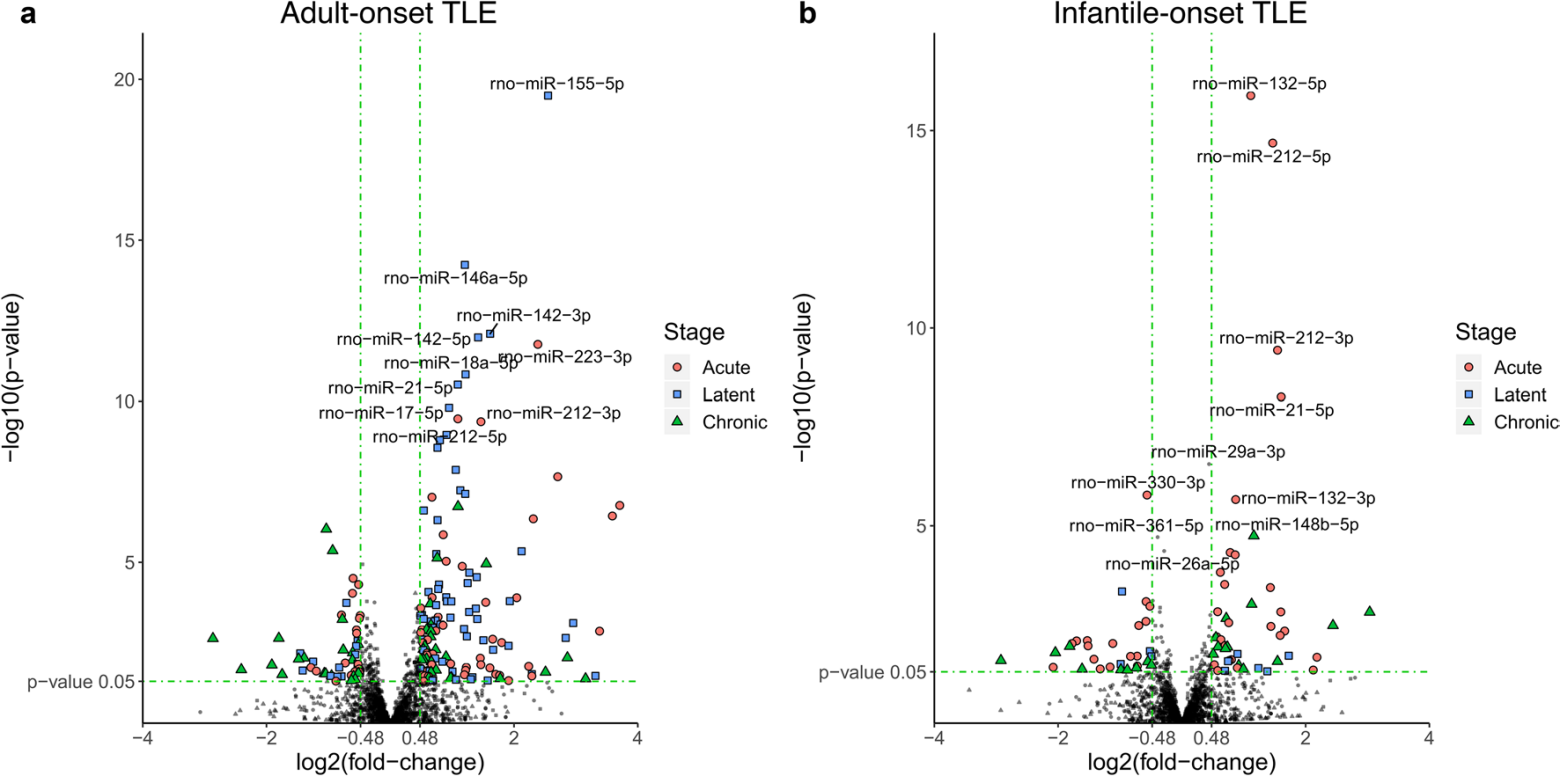
Expression changes of all miRNAs detected in adult and infantile-onset of epileptogenesis. The volcano plots display changes in miRNA expression after SE induced in adulthood (**a**) and infancy (**b**). Scattered points represent individual miRNAs identified by DESeq2. The x-axis specifies the log2 fold-changes (log2(FC)), and the y-axis specifies the negative logarithm to the base 10 of the *p*-values. Green vertical and horizontal dashed lines reflect the filtering criteria (log2(FC) =  ± 0.48 and *p*-value = 0.05). log2(FC) >  + 0.48 indicates miRNA levels increased by > 1.4 times in SE-treated rats, whereas log2(FC) <  − 0.48 indicates miRNA levels reduced by > 1.4 times in SE-treated rats. miRNAs significantly dysregulated in the acute stage of epilepsy are depicted by red circle points. miRNAs dysregulated in the latent stage are represented as blue square points, and in the chronic stage, they are represented as green triangle points. Non-significant miRNAs are depicted in black. Ten miRNAs with the highest fold-change difference between SE and control animals in adult and infantile-onset group respectively are labeled.

To address epilepsy-related alterations of miRNA expression, we employed DESeq2 and limma analyses of differential expression (comparing controls vs. post-SE animals) within each age group and epilepsy stage (Fig. 2). Both approaches identified up to 42 miRNAs with altered expression in SE animals (*p*-value < 0.05), reaching minimal fold change of 1.4 and read count over 500 in all stages of adult-onset epilepsy (Table S1A). Six of these miRNAs show altered expression across all epilepsy stages. In the infantile-onset group, the number of differentially expressed miRNAs was lower in all stages (Table S1B). None of these miRNAs was aberrantly expressed in all stages, while three miRNAs were altered in the acute and the chronic stages. Furthermore, multiple miRNAs showed altered expression in both adulthood and infantile-onset epilepsy (DESeq2: 16; limma: 17). The sequencing results of the chronic stage (of both infantile- and adult-onset TLE) were described in our publication addressing the epilepsy onset age impact on miRNA expression in chronic epilepsy in human and rat^10^.

### Validation of miRNA profiles

Although MPS is the capstone technique for nucleic acid detection, it might introduce a technology-related bias (e.g., mapping errors)^11^ requiring results to be validated by secondary methods. We reduced the bias by further expression analysis using adjusted qRT-PCR for miRNA quantification^12^. Based on MPS results, we chose a subset of miRNAs for validation (37 in adult-onset and 13 in infantile epilepsy), following the criteria: (a) the number of reads above 500; (b) *p*-value < 0.01 in both DESeq2 and limma; and (c) expression fold-change above 1.4 or below − 1.4 (Fig. 2). We added 15 and 14 miRNAs in infantile- and adult-onset epilepsy group respectively, with a *p*-value below 0.05 in at least two different stages of epilepsy or one stage within both age groups. Table S2 contains the list of miRNAs selected for miQPCR (n = 60).

We optimized miQPCR primers for the selected miRNAs following MIQE Guidelines^13^. Validation of six primers was impossible due to high GC content, T repetitions, or similarity to other high abundant miRNAs.

**Figure 3:**
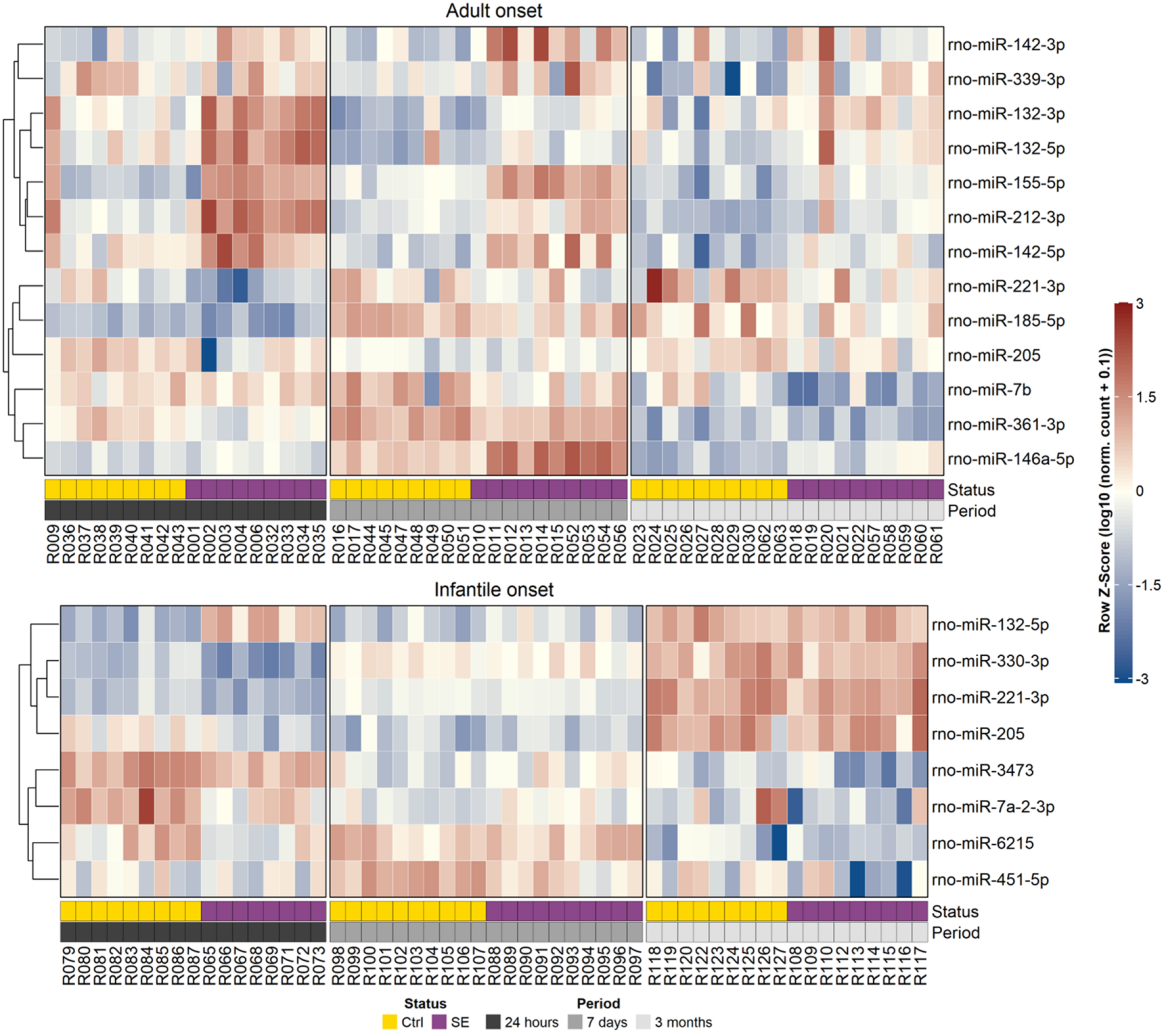
The visualization of expression and clustering analyses of dysregulated miRNAs. Heatmap shows expression profiles for PCR-validated differentially expressed miRNAs in infantile and adult-onset epileptogenesis. Rows represent individual miRNAs, while columns correspond to samples (labelled R + randomly assigned sample number) from control (Ctrl; gold) and post-status epilepticus (SE; purple) rats. Each heatmap is split into blocks representing the periods between status epilepticus (SE) induction and sample collection: 24 h (dark gray), 7 days (gray), and 3 months (light gray). The visualized expression was normalized to a row-wise Z-score (subtracting the row mean from each cell, and then dividing the value by the standard deviation of the row). The Z-score color range was further scaled to − 3 to 3 for better visualization. The initial expression values were log10(expression + 0.1) transformed prior to Z-score normalization. The miRNA clustering dendrogram is shown on the left and revealed miRNAs with similar expression patterns across collection periods.

From the subset of 54 selected miRNAs, the miQPCR validated the dysregulation of eight in infantile rats and 13 miRNAs in adult rats with fold-change over 1.4 and *p*-value < 0.5 (Table 2). This validation confirmed that infantile rats had miR-205, -221-3p, -301a-3p, -330-3p, -6215 and -7a-2-3p downregulated and miR-132-5p upregulated in the acute phase of epilepsy. No miRNA was dysregulated in the latent stage and miR-451-5p was downregulated in the chronic stage in infants. In adult rats, qPCR analysis verified that miR-361-3p was downregulated and miR-132-3p, -142-3p, -142-5p, -155-5p, and -212-3p were upregulated in the acute stage. In the latent stage, adults had miR-185-5p, and -7b downregulated and miR-146a-5p upregulated. In the chronic stage, downregulated were miR-205, -221-3p, and -339-3p, while miR-132-5p, -142-5p and -146a-5p were upregulated (Fig. 3).

**Table 2:** Differentially expressed miRNAs in infantile-onset and adult-onset TLE.

Epilepsy onset	miRNA	Acute phase				Latent phase				Chronic phase			
		miQPCR		NGS		miQPCR		NGS		miQPCR		NGS	
		*p*-value	FC	*p*-value	FC	*p*-value	FC	*p*-value	FC	*p*-value	FC	*p*-value	FC
Adult	miR-132-3p	*	1.9	**	1.8	–	1.2	**	1.4	–	− 1.2	*	1.4
	miR-132-5p	–	1.1	**	1.6	–	1.0	–	1.2	*	2.5	**	1.3
	miR-142-3p	*	2.7	**	1.9	–	1.3	**	3.1	–	1.3	*	1.5
	miR-142-5p	**	18.5	**	1.8	–	− 1.8	**	2.7	**	2.8	*	1.6
	miR-146a-5p	–	− 1.2	–	1.2	*	3.0	**	2.3	*	1.9	**	2.1
	miR-155-5p	*	6.7	**	6.5	–	1.1	**	5.9	–	− 1.2	**	2.9
	miR-185-5p	–	− 1.6	–	− 1.1	*	− 1.5	*	− 1.5	–	− 1.7	–	− 1.1
	miR-205	–	− 1.5	*	− 1.7	–	− 1.5	–	1.0	*	− 2.5	*	− 2.2
	miR-212-3p	*	1.6	**	2.8	–	− 1.0	**	1.7	–	− 1.3	**	1.7
	miR-221-3p	–	− 1.4	**	− 1.5	–	− 1.1	–	− 1.2	*	− 1.5	*	− 1.3
	miR-339-3p	–	− 1.6	–	− 1.1	–	− 1.5	*	1.3	*	− 1.9	*	− 1.5
	miR-361-3p	**	− 1.7	**	− 1.4	–	1.1	–	− 1.2	–	− 1.3	–	− 1.1
	miR-7b	–	− 1.0	–	1.0	*	− 5.0	**	− 1.5	–	− 2.1	**	− 1.9
Infantile	miR-132-5p	*	4.1	**	2.2	–	− 1.5	–	1.2	–	3.4	–	− 1.0
	miR-205	**	− 2.7	**	− 1.7	–	− 7.4	–	1.1	–	1.1	–	− 1.0
	miR-221-3p	*	− 1.4	**	− 1.3	–	− 3.3	–	− 1.1	–	1.5	–	− 1.1
	miR-330-3p	*	− 2.3	**	− 1.5	–	− 1.1	–	− 1.0	–	1.4	–	− 1.0
	miR-3473	**	− 1.9	*	− 2.7	**	− 3.4	–	1.8	–	− 1.2	–	− 1.9
	miR-451-5p	–	− 1.2	–	− 1.0	NA	NA	*	− 1.4	*	− 1.8	*	− 1.6
	miR-6215	*	− 2.1	*	− 2.7	–	− 3.2	–	− 1.2	–	− 1.5	–	1.5
	miR-7a-2-3p	*	− 3.3	**	− 1.5	–	− 3.0	–	1.2	–	− 1.7	–	− 1.4

List of miRNAs with altered regulation (fold-change over 1.4) confirmed by miRNA sequencing (DESeq2) and miQPCR. 13 miRNAs differentially expressed in adulthood onset epilepsy (six in acute, three in latent and six in chronic stage) with one miRNA common to the acute and chronic stage and two for the latent and chronic stage. Eight miRNA in total altered in animals with epileptic status at P12: seven altered in the acute stage and one in the chronic stage.

**p-value < 0.01; *0.01 < *p*-value < 0.05; – *p*-value > 0.05; *NA* not identified, *FC* fold change.

We used miQPCR to quantify the expression of miRNAs included in validation through all epilepsy stages within the respective age group, even if MPS detected their dysregulation in a single stage only. In these cases, miQPCR confirmed the non-significant results of MPS. miR-3473 was identified as downregulated (*p* = 0.00) only by miQPCR in the latent stage of childhood epilepsy.

### Putative targets of miRNAs dysregulated in epilepsy

We used the *MirTarget* search tool^14^ to make a list of predicted brain-function related targets with a target score above 90 for each miRNA included in the miQPCR validation group respectively (Table S3). Among predicted targets of 15 miRNAs are subunits of ion channels (calcium, chloride, potassium, sodium) while seven miRNAs regulate receptors of γ-aminobutyric acid (GABA) and glutamate. The largest group of predicted targets consists of solute carrier transporters regulated by 20 miRNAs. *miRTarbase* database provides information about experimentally validated targets of individual miRNAs^15^. 16 differentially expressed miRNAs detected by MPS (38%) have at least one validated target that is expressed in rat nervous system. Among these targets stood out forehead box O3 (*Foxo3*) targeted by three miRNAs. Dysregulated miRNAs confirmed by PCR validation regulate altogether 19 experimentally validated targets based on *miRTarbase* data (Table S3). Figure S2 displays these putative or validated targets of dysregulated miRNAs in the interactome form with regard to the epilepsy stage and the age at SE induction.

We employed the *DIANA-mirPath* v3 to identify potential targets and pathways affected by miRNAs with altered expression validated by MPS and miQPCR^16^. The *DIANA-mirPath* tool identified seven pathways potentially affected by miRNAs with altered levels in infant rats 24 h after SE (Table 3). This table includes pathways involved in cell cycle and communication, which share multiple genes (Long-term depression, TGF-beta, Hippo, and Wnt signalling) and excitatory/ inhibitory neurotransmitter pathways (GABAergic and Glutamatergic signalling). In the chronic stage of infantile-onset epilepsy, *DIANA-mirPath* identified two gene targets of miR-451-5p (*Prps2*—Phosphoribosyl Pyrophosphate Synthetase 2 and *Gfpt2*—Glutamine-Fructose-6-Phosphate Transaminase 2) involved in multiple metabolic pathways.

**Table 3:** Predicted pathways and genes affected by aberrantly expressed miRNAs in TLE with onset in adulthood and infancy.

	Pathway	miRNAs involved	Genes affected	
**Predicted targets in infantile-onset epilepsy**				
Acute	TGF-β signalling	miR-205, -330-3p,-3473, -7a-2-3p	*Smad1*	SMAD family member 1
			*Smad5*	SMAD family member 5
			*Smad7*	SMAD family member 7
			*Bambi*	BMP and activin membrane-bound inhibitor
			*Id2*	Inhibitor of DNA binding 2, HLH protein
			*Zfyve16*	Zinc finger FYVE-type containing protein 16
			*Ppp2ca*	Serine/threonine-protein phosphatase 2CA catalytic subunit alpha isoform
			*Acvr1*	Activin A receptor type 1
			*Smurf2*	SMAD specific E3 ubiquitin protein ligase 2
	Morphine addiction	miR-205, -221-3p, -330-3p	*Gabra1*	Gamma-aminobutyric acid type A receptor gamma 1 subunit
			*Gnb3*	Guanine nucleotide-binding protein G(I)/G(S)/G(O) subunit gamma-3
			*Pde3b*	Phosphodiesterase 3B
	Long-term depression	miR-205, -221-3p, -330-3p,-7a-2-3p	*Gnai2*	Guanine nucleotide-binding protein G(s) subunit alpha
			*Itpr2*	Inositol 1,4,5-trisphosphate receptor, type 1
			*Ppp2ca*	Serine/threonine-protein phosphatase 2CA catalytic subunit alpha isoform
	Hippo signalling	miR-205, -221-3p, -330-3p,-7a-2-3p	*Smad1*	SMAD family member 1
			*Smad7*	SMAD family member 7
			*Ppp2ca*	Serine/threonine-protein phosphatase 2CA catalytic subunit alpha isoform
			*Ywhag*	Tyrosine 3-monooxygenase/tryptophan 5-monooxygenase activation protein, eta
			*Id2*	Inhibitor of DNA binding 2, HLH protein
			*Prkci*	Protein kinase C, iota
			*Axin2*	Axin 2
			*Tef7l2*	Lymphoid enhancer binding factor 2
			*Dlg2*	Discs large MAGUK scaffold protein 1
	Wnt signalling	miR-205, -221-3p, -330-3p,-6215, -7a-2-3p	*Bambi*	BMP and activin membrane-bound inhibitor
			*Tbl1xr1*	Transducin (beta)-like 1 X-linked
			*Nfatc3*	Nuclear factor of activated T-cells 3
			*Ppp3r1*	Protein phosphatase 3, regulatory subunit B, alpha
			*Prickle2*	Prickle planar cell polarity protein 2
			*Cxxc4*	CXXC finger protein 4
			*Axin2*	Axin 2
			*Siaha*	Siah E3 ubiquitin protein ligase 1
			*Sox17*	Transcription factor SOX17
			*Tef7l2*	Lymphoid enhancer binding factor 2
			*Nlk*	Nemo like kinase
	Glutamatergic synapse	miR-205, -221-3p, -330-3p,-6215	*Gnb3*	Guanine nucleotide-binding protein G(I)/G(S)/G(O) subunit gamma-3
			*Gnai2*	G protein subunit alpha i2
			*Ppp3r1*	Protein phosphatase 3, regulatory subunit B, alpha
			*Itpr2*	Inositol 1,4,5-trisphosphate receptor, type 1
			*Homer2*	Homer scaffolding protein 1
	GABAergic synapse	miR -205, -221-3p, -330-3p	*Gabra1*	Gamma-aminobutyric acid type A receptor gamma 1 subunit
			*Gnb3*	Guanine nucleotide-binding protein G(I)/G(S)/G(O) subunit gamma-3
			*Trak2*	Trafficking kinesin protein 2
Chronic	Pentose phosphate pathway	miR-451-5p	*Prps2*	Phosphoribosyl pyrophosphate synthetase 2
	Glutamate metabolism	miR-451-5p	*Gfpt2*	Glutamine fructose-6-phosphate transaminase 2
	Sugar metabolism	miR-451-5p	*Gfpt2*	Glutamine fructose-6-phosphate transaminase 2
	Biosynthesis of amino acids	miR-451-5p	*Prps2*	Phosphoribosyl pyrophosphate synthetase 2
	Carbon metabolism	miR-451-5p	*Prps2*	Phosphoribosyl pyrophosphate synthetase 2
**Predicted targets in adult-onset epilepsy**				
Acute	MAPK signalling	miR**-132-3p,** -142-3p, -142-5p, -155-5p, **-212-3p**, -361-3p	*Cacnb4*	Calcium voltage-gated channel auxiliary subunit beta 4
			*DUSP2 And 9*	Dual specificity phosphatase 2 and 9
			*Fgf14*	Fibroblast growth factor 4
			*Il1a*	Interleukin 1 alpha
			*MAP3K3, 6 And 14*	Mitogen activated protein kinase 3, 6 and 14
			*Mapkapk2*	Mitogen-activated protein kinase-activated protein kinase 2
			*Mecom*	Ecotropic virus integration site 1 protein
			*Nlk*	nemo like kinase
			*Ptpn5*	Protein tyrosine phosphatase, non-receptor type 5
			*Rap1b*	Ras-related protein Rap-1B
			***Rasa1***	**RAS P21 Protein Activator 1**
			*Rasgrf1*	RAS protein-specific guanine nucleotide-releasing factor 2
			*Rela*	RELA proto-oncogene, NF-kB subunit
			*RPS6KA3 And 4*	Ribosomal protein S6 kinase A3 and 4
			*RRAS Adn 2*	Ras-related protein R-Ras
			*Sos1*	SOS Ras/Rac guanine nucleotide exchange factor 1
			*Srf*	Serum response factor
			*Tab**2*	TGF-beta activated kinase 1/MAP3K7 binding protein 2
			*Tgfbr1*	Transforming growth factor, beta receptor 1
	Ras signalling	miR**-132-3p**, -142-3p, -142-5p, -155-5p, **-212-3p**, -361-3p	***Bcl2l1***	**Bcl-2-like 1 (apoptosis regulator Bcl-X)**
			*Csf1r*	Colony stimulating factor 1 receptor
			*ETS1 And 2*	ETS proto-oncogene 1 and 2, transcription factor
			*Foxo4*	Forkhead box protein O4
			*Gab1*	GRB2-associated binding protein 1
			***Grin2a***	**Glutamate Ionotropic Receptor NMDA Type Subunit 2A**
			*Kitlg*	KIT ligand
			*Rap1b*	Ras-related protein Rap-1B
			***Rasa1***	**RAS P21 Protein Activator 1**
			*Rasgrf1*	RAS protein-specific guanine nucleotide-releasing factor 1
			*Rela*	RELA proto-oncogene, NF-kB subunit
			*Rgl2*	Ral guanine nucleotide dissociation stimulator-like 2
			*RRAS And 2*	Ras-related protein R-Ras
Latent	Mucin type O-glycan biosynthesis	miR-185-5p, -7b	*Galnt3*	Polypeptide N-acetylgalactosaminyltransferase3
			*Galnt5*	Polypeptide N-acetylgalactosaminyltransferase5
	Synaptic vesicle cycle	miR-185-5p, -7b	*Syt1*	Synaptotagmin 1, isoform A
			*Snap25*	Synaptosomal-associated protein 25
			*Slc18a2*	Solute carrier family 18 member 2
			*Atp6v1e1*	ATPase H + transporting V1 subunit E1
Chronic	Amphetamine addiction	miR-142-5p,-146a-5p, -221-3p	*Ddc*	Dopa decarboxylase
			*Fos*	FBJ osteosarcoma oncogene
			*Ppp3r1*	Protein phosphatase 3, regulatory subunit B, alpha
			*Slc18a2*	Solute carrier family 18 (vesicular amine transporter), member 2
	Cocaine addiction	miR-142-5p**,-146a-5p**, -221-3p	*Dlg4*	Discs large MAGUK scaffold protein 4
			*Gnai2*	G protein subunit alpha i2
			*Ddc*	Dopa decarboxylase
			***Nfkb1***	**Nuclear Factor Kappa B Subunit 1**
			*Slc18a2*	Solute carrier family 18 (vesicular amine transporter), member 2
	GABAergic synapse	miR-146a-5p, -205, -221-3p	*Gabra1*	Gamma-aminobutyric acid type A receptor gamma 1 subunit
			*Gnb3*	Guanine nucleotide-binding protein G(I)/G(S)/G(O) subunit gamma-3
			*Trak2*	Trafficking kinesin protein 2
			*Scl38a5*	Solute carrier family 38 (sodium-coupled neutral amino acid transporter), member 5
	Hippo signalling	miR-205, -221-3p, -330-3p,-142-5p**, -146a-5p**	*Smad1*	SMAD family member 1
			***Smad4***	**SMAD family member 4**
			*Smad7*	SMAD family member 7
			*Ywhag*	Tyrosine 3-monooxygenase/tryptophan 5-monooxygenase activation protein, eta
			*Id2*	Inhibitor of DNA binding 2, HLH protein
			*Axin2*	Axin 2
			*Tef7l2*	Lymphoid enhancer binding factor 1
			*DLG2 And 4*	Discs large MAGUK scaffold protein 2 and 4
	Morphine addiction	miR-142-5p, -205, -221-3p	*Gabra1*	Gamma-aminobutyric acid type A receptor gamma 1 subunit
			*Gnb3*	Guanine nucleotide-binding protein G(I)/G(S)/G(O) subunit gamma-3
			*Pde3b*	Phosphodiesterase 3B
	NF-κB signalling	miR**-146a-5p**, -221-3p	***Traf6***	**TNF receptor associated factor 6**
			*Map3k14*	Mitogen-activated protein kinase kinase 14
			***Nfkb1***	***Nuclear Factor Kappa B Subunit 1***
			***Irak1***	**Interleukin-1 receptor-associated kinase 4**
	TGF-β signalling	miR-205, -142-5p**, -146a-5p**	*Smad1*	SMAD family member 1
			***Smad4***	**SMAD family member 4**
			*Smad7*	SMAD family member 7
			*Bambi*	BMP and activin membrane-bound inhibitor
			*Id2*	Inhibitor of DNA binding 2, HLH protein
			*Zfyve16*	Zinc finger FYVE-type containing protein 16
	Ubiquitin mediated proteolysis	miR-205, -221-3p,-142-5p, **-146a-5p**	*Herc3*	E3 ubiquitin-protein ligase HERC3
			*Mgrn1*	E3 ubiquitin-protein ligase MGRN1
			*Siah1a*	Siah E3 ubiquitin protein ligase 1
			***Traf6***	**TNF receptor associated factor 6**
			*UBE2a,2e3,3A, 4A*	Ubiquitin-conjugating enzyme E2A, 2E3, 3A, 4A
			*Uba 6*	Ubiquitin-like modifier activating enzyme 6
			*Wwp1*	WW domain containing E3 ubiquitin protein ligase 1
	Wnt signalling	miR-205, -221-3p, -330-3p,-6215, -7a-2-3p	*Bambi*	BMP and activin membrane-bound inhibitor
			*Tbl1xr1*	Transducin (beta)-like 1 X-linked
			*Nfatc3*	Nuclear factor of activated T-cells 3
			*Ppp3r1*	Protein phosphatase 3, regulatory subunit B, alpha
			*Cxxc4*	CXXC finger protein 4
			*Axin2*	Axin 2
			*Siah1a*	Siah E3 ubiquitin protein ligase 1
			*Nlk*	Nemo like kinase
			*Vangl1*	VANGL planar cell polarity protein 2
			*Fzd1*	Frizzled 1/7

Detailed list of predicted pathways and genes regulated by miRNAs with altered expression across three epilepsy stages in TLE model. *DIANA mirPath v3* software generated predicted pathways based on miRNA lists uploaded for each epilepsy stage with both onset groups analysed separately. Bold font marks the miRNAs and their targets validated in rat included in *miRTarbase* database.

miRNAs dysregulated after SE in adult animals putatively affect multiple pathways across all three stages of epileptogenesis (Table 3). The majority of affected pathways involved immune response, cell proliferation, differentiation, and apoptosis (Wnt, TGF-β, NF-κB, Ras and MAPK signalling, etc.). Furthermore, the synaptic vesicle cycle is among predicted pathways in the latent epilepsy stage while GABAergic synapse was identified in the chronic stage. Several miRNA-gene pairs (such as miR-132-3p/-212-3p and RAS P21 Protein Activator 1) occurring repeatedly in these pathways were already validated in rats and listed in *miRTarbase* database (Table 3B).

## Discussion

Despite the growing evidence of miRNA involvement in epilepsy, knowledge about the relationship between miRNA expression and TLE onset in the developing brain is limited. The uniqueness of this study lays within the comparison of the whole miRNome throughout three stages of the infantile- and adult-onset TLE. Our large-scale screening discovered 23 miRNAs likely to participate in epileptogenesis, while the majority of them is in concordance with published data^5^. Applying two quantitative methods (MPS and miQPCR), we enhanced the reliability of our result showing that acute, latent, and chronic stages of TLE have unique miRNA imprints affected by the age of epilepsy onset. Finally, target prediction linked these alterations in miRNA profiles with genes and biological pathways implied in epileptogenesis.

Massive parallel sequencing (MPS) offers the complete evaluation of all small RNAs^11^, but the literature recommends combined use of the methods analysing differential expression (DE) to reduce technology-related bias. Thus, we employed two DE analyses (DESeq2 and limma), which produced similar results of miRNA detection and validated sequencing results using miQPCR^12^. The cross-platform comparison did not confirm the significance of all miRNAs identified by MPS. This incomplete concordance might result from the pre-amplification and relaxed mapping parameters in the MPS introducing false-positive miRNA detection^17^. Besides, the majority of miRNAs not validated by miQPCR had low abundance in samples (their Ct values were close to the background noise), which could distort the quantification, causing the discrepancy between used methods. Overall, the application of two DE analyses of MPS data and additional validation of selected miRNAs increased the reliability of our results.

Since their introduction to epilepsy in the year 2010, miRNAs have become vitally studied in both patients and animal models of epilepsy^5,18^*.* However, the majority of animal studies focus solely on miRNA profiling in animal models of adult-onset epilepsy without considering the onset age. Here, we addressed the miRNA profile in animals with SE induced at postnatal day 12, which corresponds to infancy in human^19^. Patients with early-onset TLE are at higher risk of developing: structural changes in the brain, resistance to treatment by anti-epileptic drugs, cognitive impairment, and psychiatric comorbidities^2,20^. Mathern and colleagues reported that initial precipitating injuries that lead to aggravated TLE have high incidence before the first year of age^20^. The outcome of infantile- and adult-onset epilepsy also differs in animal models. In infantile-onset Li/pilocarpine-induced epilepsy, animals suffer from less severe brain damage located mainly in the hippocampal area, amygdala, and mediodorsal thalamus^21,22^. The development of epilepsy is gradual and milder in both spontaneous seizures (electrographic) and cognitive and behavioural impairment, which present early on^8,23^.

Early-onset epilepsy has been linked with changes in miRNA profiles in five studies so far. Four examined pre-defined groups of miRNAs in rats with epilepsy-induced on a postnatal day 25 or later and/or children^24–27^ while Omran and colleagues focused on miR-146a in P12 rats^28^. In this study, miRNA sequencing enabled comparison of the whole miRNome between the infantile and adult-onset TLE. In adult-onset TLE, five miRNAs showed an increase after SE in the acute stage while only one (miR-361-3p) was decreased (Fig. 2). Three of the miRNAs dysregulated in the chronic stage of adult-onset epilepsy (miR-132-5p, -205 and -211-3p) exhibit dysregulation with the same trend in the acute stage of epilepsy in infant rats. Altogether, SE in the immature brain induces changes in the expression of fewer miRNAs across all stages. Seven miRNAs show altered expression in the acute stage of the disease in young animals, which might be critical for brain development. Interestingly, all miRNAs but miR-132-5p were downregulated 24 h after SE induced in P12, suggesting different mechanisms involved in the acute response to SE in the immature brain. In our recent study, we have shown that chronic miRNA profile depends on the epilepsy onset age in both rats and humans, while the adult-onset epilepsy model shows greater overlap with patients with refractory epilepsy compared with the infantile-one^10^.

Our results are consistent with published data: altered expression of 14 miRNAs was previously reported in the same stage of epilepsy in animal models (Table S4). Even though miRNA profiles of animal models of TLE are often contradictory and do not overlap with human data^29^, miR-132-3p, -132-5p, -142-3p, -142-5p, -146a-5p, -155-5p, -221-3p and -330-3p show altered expression in both our model and TLE patients^7,26,30,31^. The only miRNAs with the opposite trend of dysregulation from patients are miR-361-3p and -451-5p^7,31,32^, while miR-3473 and -6215 have not been associated with epilepsy so far.

The role of multiple validated miRNAs in epilepsy has been scrutinized in animal models and cell cultures. Our results are in concordance with Aronica and colleagues, who reported that the expression of miR-146a-5p is elevated in the latent and the chronic stages of TLE in adult rats and patients^18^. They have suggested a mechanism of miRNA-146a-5p action involving interleukin 1β (IL-1β), nuclear factor kappa-B (NFkB), toll-like receptor (TLR) and tumour necrosis factor α (TNF-α) pathways. This mechanism was confirmed by Omran and colleagues, who showed a negative correlation between miR-146a-5p and these factors^28,33^. They have further observed that inhibition of miR-146a-5p prior to SE worsens its outcome, while miR-146a-5p mimic shows a protective effect.

Moreover, Ashhab and colleagues observed positive correlations of TNF-α with miR-155-5p in immature TLE rats (SE induced in P25) and TLE children, as well as astrocyte cultures^30^. We have detected miR-155-5p upregulation in MPS analysis of the acute stage in immature rats; however, this miRNA did not reach statistical significance in miQPCR. On the other hand, we validated the altered expression of this miRNA in the acute stage of TLE in adulthood.

miRNA-132-3p mediates synaptic plasticity and dendritic growth via repression of GTPase-activating protein, p250GAP^34,35^. This miRNA also regulates the cholinergic signalling pathway through acetylcholinesterase^36^. Injection of miR-132-3p antagonist 24 h before SE reduces seizure-induced neuronal death in kainic acid mice^37^. Interestingly, we have observed an increase in expression of miR-132-5p in both adult- and infantile-onset TLE, however, its function in the brain remains to be studied.

miR-142-5p targets mitochondrial Rho GTPase 1(Miro1 protein), and mitochondrial trafficking kinesins (Trak1 and 2). Inhibition of miR-142-5p attenuates pilocarpine-induced SE in mice and reduces neuronal death^38^. miR-142-5p cooperates with miR-10a-5p, miR-21a-5p on regulation of TGF-β signalling and their co-inhibition shows anti-seizure effect^39^.

The majority of the discovered miRNAs with altered expression in our study has been previously described as the regulators of the tumour microenvironment and immune response in various types of cancers (e.g. miR-142–3/5p, miR-205, or miR-330-5p)^40–42^, or the modulators of immune response in rheumatoid arthritis (e.g. miR-146a)^43^. Moreover, some of them (e.g., miR-132, miR-146a, miR-155-5p or miR-212-3p) have been previously associated with other neurological diseases (such as Parkinson`s or Alzheimer` diseases)^40,44–46^ and miR-212-3p has been suggested as a blood biomarker of Alzheimer’s Disease^44^. Since our discovered miRNAs have been previously connected with other neurological disorders, our findings support the tight connection of these miRNAs with the brain microenvironment.

Fine-tuned miRNA regulation is essential for proper brain function and development. Hence, we scrutinized putative targets of miRNAs dysregulated during epileptogenesis using target prediction tools (*MirTarget*, *DIANA-mirPath v3*). Potassium channels-considered as potential initiators of epilepsy^47^-occurred among putative targets of five miRNAs with altered expression in infantile and eight miRNAs in adult-onset epilepsy. Potassium channels may contribute to the electrochemical imbalance in neurons along with other ion channels (chloride, sodium, calcium) scoring among targets of dysregulated miRNAs. Another target related to neuronal excitability is the most important inhibitory neurotransmitter-γ-aminobutyric acid (GABA), often associated with epilepsy^47^. Based on our analysis, miRNAs seem to affect GABAergic receptors in the chronic stage of adult-onset and the acute stage of infantile-onset epilepsy. Interestingly, our data show that miRNA expression imbalance in the acute stage of epilepsy in the developing brain might also modify a major excitatory neurotransmitter pathway-glutamatergic signalling^48^. This suggests complex epilepsy-induced changes in the regulation of neuronal excitability during brain development (Table 3A).

Excitability might also increase as a consequence of dysregulation of inflammatory pathways, cell cycle, differentiation, and apoptosis^49^. These processes are regulated by major signal transduction pathways (Wnt, TGF-β, MAPK, Ras signalling, etc.) identified as putative targets of dysregulated miRNAs in both age groups Several of these targets were already experimentally validated in rat tissues (Table 3A and B). Wnt, Ras, and MAPK signalling pathways increase seizure susceptibility in animal models of epilepsy and cell cultures^50–52^ while TGF-β blockers prevent from epileptogenesis in blood–brain barrier breakdown models^53^. Furthermore, MAPK signalling induces mossy fibre sprouting in rats after traumatic brain injury^54^. All of these pathways contribute to the complex pathology of epilepsy and might serve as targets for future miRNA-therapy. Nevertheless, the relationship between these pathways and miRNAs in epilepsy needs to be confirmed.

**Figure 4:**
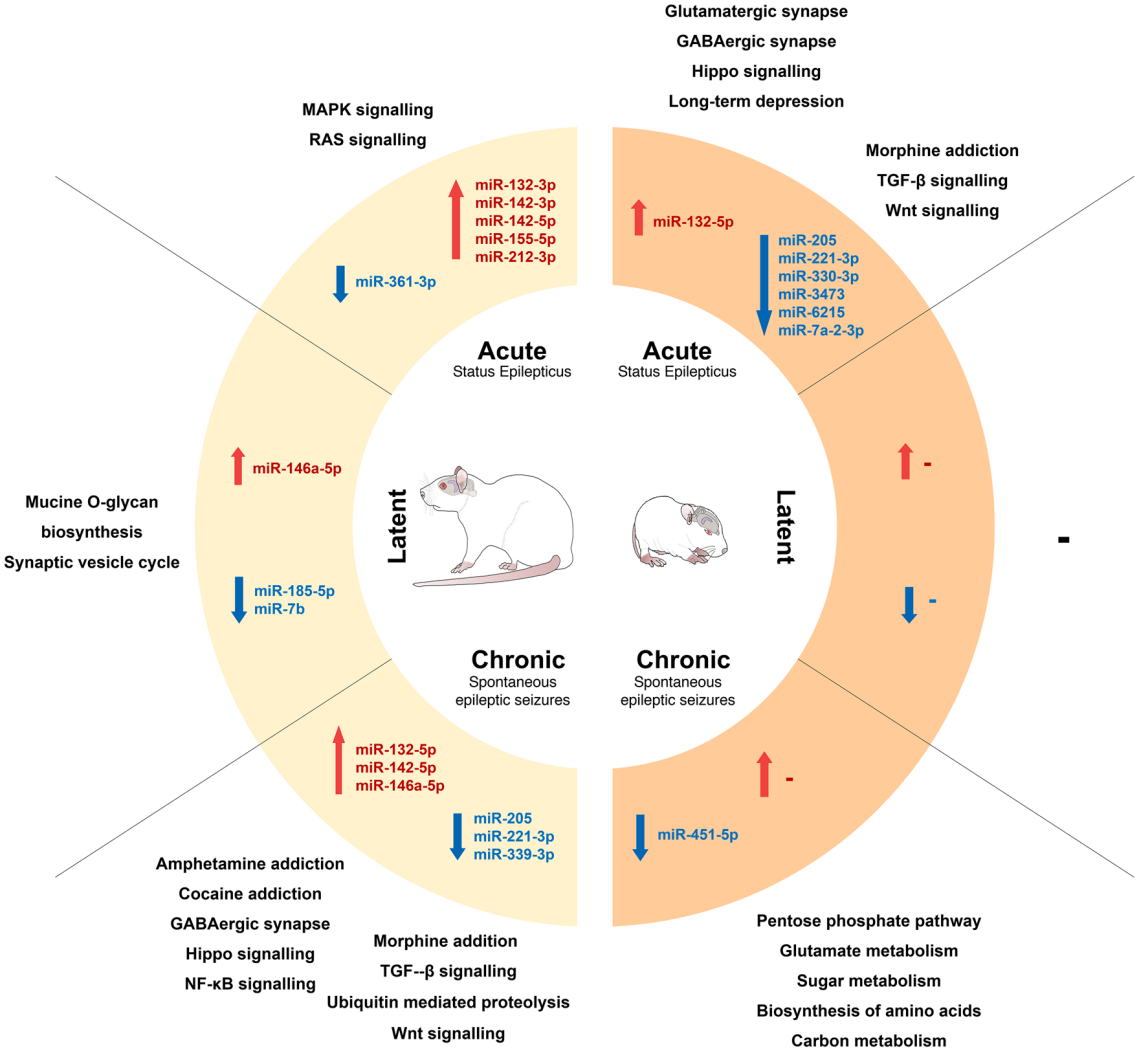
miRNAs with altered expression in adult and infantile-onset epilepsy-summary diagram. The diagram summarizes the occurrence of dysregulated miRNAs after status epilepticus (SE) induced in adulthood (left; adult rat) and infancy (right; rat pup). The inner parts specify the stage of epileptogenesis with a short description of seizure occurrence. The middle parts contain the lists of dysregulated miRNAs in the respective stages. Upregulated miRNAs are displayed in red, while downregulated miRNA after SE are shown in blue. The outer parts list the predicted pathways affected by dysregulated miRNAs in the given stage.

In conclusion, we have identified and validated differential expression of miRNAs that are likely to play a role in adult- and infantile-onset TLE (Fig. 4). Despite the similarities, epilepsy onset in the immature brain induces specific alterations in the miRNA profile with a high prevalence of miRNA downregulation. Electrographic seizures typical for the chronic stage in the pilocarpine model of infantile-onset TLE are accompanied by dysregulation in a lower number of miRNAs than motoric seizures in adult animals. Awareness of these age-induced differences in miRNA expression is essential for understanding the miRNA to the pathology of epilepsy and the timing of miRNA therapy. miRNAs dysregulated in each epilepsy onset age were associated with pathways involved in epilepsy via analysis of putative targets. Further examination of these pathways and their altered miRNA regulation may explain the aggravating features of infantile-onset epilepsy and novel targets for medical interventions.

## Materials and methods

### Animals

Male Wistar albino rats aged P12 (P- postnatal day; n = 62) or P60 (n = 83) (Institute of Physiology, Czech Academy of Sciences, Prague) were used in this study. The day of birth was defined as day 0, and the animals were weaned on P28. Rats were housed in a controlled environment (temperature 22 ± 1 °C, humidity 50% to 60%, 12 h light cycle), with free access to food and water. All procedures involving animals and their care were conducted according to the ARRIVE guidelines^55^. Procedures for this study complied with the regulations covering animal experimentation within the European Union (European Communities Council Directive 86/609/EEC) and the Czech Republic (Act No 246/1992 Coll.). The Ethical Committee of the Czech Academy of Sciences approved the experimental protocol (Approval No. 128/2013).

### Induction of epileptic status

Status epilepticus (SE) was induced as described previously^8^. Briefly, all animals were administered with LiCl (127 mg/kg; # L-0505, Sigma Chemical Co.) 24 h hours prior to pilocarpine/saline injection. Animals were randomly assigned to control and status epilepticus (SE) groups. SE was induced by a single intraperitoneal dose of pilocarpine (35 mg/ml/kg in P12 and 45 mg/ml/kg in P60; # P-6503, Sigma Chemical Co.) in the SE group of animals at P12 or P60. After pilocarpine injection, animals were caged separately and continually observed for three hours. One and half hours after the onset of convulsive SE, animals were injected with a single intraperitoneal dose of paraldehyde (0.07 ml/kg and 0.6 ml/kg, respectively; # 76260, Fluka Chemie AG, Buchs, Switzerland) to decrease mortality. Animals injected at P12 were placed on a heating pad set to 32–33 °C throughout the whole procedure. Only rats that exhibited behavioural manifestations of seizures progressing to forelimbs clonus for at least one hour were used for further studies. For more details, see Supplementary methods.

### Monitoring

Animals assigned to a 3-month interval were observed continuously through uninterrupted video-monitoring for 1 week before scarifying with IP infrared Camera Edimax IC-3140 W for wireless monitoring. *Synology Surveillance Station 7* software was used for both registration and evaluation. Recordings were evaluated manually by an experienced observer. The incidence of motor seizures (Racine stage 3–5) was registered. The electrographic analysis was omitted in this study to prevent possible inflammation arising from the implantation of EEG electrodes that might alter the miRNA profile.

### Hippocampal tissue collection and RNA isolation

Hippocampal tissue was collected in both age groups at three stages of epileptogenesis: (a) acute (24 h); (b) latent (7 days); and (c) chronic (3 months after SE). Both age groups consisted of 10 SE and 10 control animals per stage. Animals were sacrificed by decapitation under an overdose of anaesthesia (ether). The brains were dissected, and the entire hippocampus was collected from both hemispheres. Tissues were immediately frozen in dry ice and stored at -80 °C until further processing. Total RNA was isolated from hippocampal tissue using the TRI Reagent® (#TR 118/200, iBiotech) according to the manufacturer’s protocol. Ceramic beads were used for tissue disruption. RNA was extracted from all specimens and quantified using Qubit® 2.0 Fluorometer (Thermo Fisher Scientific).

### Massive parallel sequencing

Sequencing libraries were prepared from 1.1 µg of total RNA obtained from 10 SE and 10 control animals per time point in both age groups (n = 120), using a NEXTflex Small RNA-Seq Kit v3 (#NOVA-5132–06, Bioo Scientific) according to the manufacturer’s protocol. After the amplification, samples were analyzed using Fragment Analyzer (Advanced Analytical), and specific fragments of the mature miRNA library (145 bp) were quantified. Samples were equally pooled based on the concentration of miRNA fragments, which were isolated by the Pippin Prep instrument using the 3% Agarose (Sage Science) prior to sequencing. Isolated fragments were quantified using Qubit™ dsDNA HS Assay Kit on Qubit® 2.0 Fluorometer (Thermo Fisher Scientific) and used for sequencing on NextSeq500 (Illumina) according to the manufacturer’s protocol.

### MPS-data processing and differential expression analysis of miRNAs

Two separate workflows were used to analyse MPS data. Adaptor sequences were scanned and identified by Kraken package (v15-065)^56^ and removed with Cutadapt (v1.12) software^57^. Only high-quality reads (Phred ≥ 10 over at least 85% of the read length) with a length between 16 and 28 bp after adapter trimming were retained as potential miRNA reads. The quality of both raw and processed reads was evaluated using FastQC software^58^. FastQ Screen (v0.10.0) and Bowtie (v1.1.2)^59^ were used to determine and remove contaminants. The raw miRNA expression levels were quantified by seqBuster (1.2.4a6) (maximum of 1 mismatch) with miRBase annotation (v22)^60^. Samples with a total number of aligned miRNA reads above 300 000 were used for evaluation of differential expression by R package DESeq2 (1.16.1)^61^. Alternatively, count-based miRNA expression data were generated by Chimira using miRBase v22 as a reference. Up to two mismatches per read were allowed during alignment. Samples with a total number of aligned miRNA reads above 500,000 were further analysed using R/Bioconductor packages^62^. Linear model fitting and an eBayes approach from the limma package assessed differential expression. Individual workflows are referred to further in the text as DESeq2 and limma based on differential expression analysis. The obtained *p*-values were adjusted for multiple testing using the Benjamini–Hochberg method. To reduce the false-positive detection we confirmed the results with qPCR quantification. Raw data and annotated sequences of the small RNA libraries that support our findings are available in the GEO database (accession number GSE124332).

### MicroRNA expression analysis using real-time PCR (miQPCR)

We applied a small RNA-specific reverse transcription method followed by miQPCR^12^ to quantify miRNAs. Primers were either designed manually or downloaded from the list of miQPCR primers^12^, validated, and optimized according to the MIQE Guidelines^13^. The sequences and melting temperatures of all validated primers are summarized in Table S5.

Selected miRNAs were quantified in parallel across all samples using the SmartChip MSND system and SmartChip Real-Time PCR Cycler (Takara). Following protocol was used for amplification: 95 °C for 5 min, followed by 45 cycles of 95 °C for 10 s, 60–62 °C for 10 s, and 72 °C for 10 s. The Ct values were calculated by SmartChip qPCR Software using the ’second derivate maximum’ method. Ct values ranged from 20 to 32, and values over 32 were considered background noise. miRNA expression was determined in tissue samples from all 120 rats in four technical replicates.

The relative transcription of the selected miRNAs was calculated from Ct values using the 2^−ΔΔ*CT*^ method^63^. RNU6B served as the endogenous control for normalization. miRNA levels between SE rats and controls were compared using the Mann–Whitney U test. Differential expression was considered statistically significant if the *p*-value was below 0.05 and fold-change was over 1.4 in SE or control samples.

### In silico miRNA target prediction

A *MirTarget* tool^14^ generated a list of predicted target genes regulated by miRNAs with differential expression in post-SE rats compared to controls identified by MPS. DIANA-mirPath v3 generated a list of putative pathways affected by miRNAs identified by MPS and validated by qPCR as dysregulated in rats after SE. Pathway prediction was based on predicted mRNA targets and experimentally validated miRNA interactions from *DIANA-TarBase*^16^. Validated targets of dysregulated miRNAs were explored using miRTarbse^15^ database and further refined for those validated with strong evidence (reporter assay or western blot) and expressed in rat nervous tissue using Rat Genome Database^64^*.* All databases (*MirTarget, DIANA-mirPath and miRTarbase*) analysed specific miRNA targets for *Rattus norvegicus*.

## Supplementary Information

Supplementary Information 1.

## Data Availability

The datasets generated during and analysed during the current study—namely raw data and annotated sequences of the small RNA libraries are available in the GEO database (Accession Number GSE124332).
