# Supplementary material for: Dynamic miRNA changes during the process of epileptogenesis in an infantile and adult-onset model
Source: Sci Rep. 2021 May 6;11:9649. doi: 10.1038/s41598-021-89084-9 (PMC8102630; doi:10.1038/s41598-021-89084-9)
Supplement: Supplementary file 1 — Supplementary Information 1. [file 41598_2021_89084_MOESM1_ESM.pdf]

Dynamic miRNA changes during the process of epileptogenesis in an infantile and adult-onset model

Bencurova Petra[1,2], Baloun Jiri[1], Hynst Jakub[1], Oppelt Jan[1], Kubova Hana[3], Pospisilova Sarka [1*], Brazdil Milan[1,2]

[1] CEITEC - Central European Institute of Technology, Masaryk University, Kamenice 5, 625 00 Brno, Czech Republic

[2] Brno Epilepsy Center, Department of Neurology, St. Anne's University Hospital and Medical Faculty of Masaryk University, Pekarska 53, 656 91 Brno, Czech Republic

[3] Academy of Sciences Czech Republic, Institute of Physiology, Department of Developmental Epileptology, Videnska 1083, 14220 Prague, Czech Republic

Supplementary Material

Supplementary methods

Induction of status epilepticus (SE) and monitoring

Two age groups (adult (P60) and twelve days old (P12)) of male Wistar albino rats (Institute of Physiology, Czech Academy of Sciences, Prague; No. of approval 1396/2014-MZE-17214) were included in this study. Healthy naïve animals with a normal weight corresponding to the age of animals were used in the experiments. Animals were maintained under controlled temperature (22±1°C) and humidity (50 to 60%) with a 12/12 h light/dark cycle (lights on at 6:00 AM.). Food and water were provided ad libitum. On day 5, (birth counted as day 0) the pups were randomly fostered by the breeder, and each litter was adjusted to ten males. Animals were weaned at postnatal day (P) 28.

For experiments with adults, groups of 10-15 P60 animals were used for this study. Each group consisted of animals born within one week with a mean weight of 369.2± 34.7g at P60. Animals were transferred from breeding facilities one week before SE induction and placed in our animal room for adaptation. On the day of SE induction animals were randomly divided into control and SE groups and assigned a code that allowed for their individual history to be followed throughout the entire study. After adaptation, all animals were given an intraperitoneal (ip.) injection of LiCl. Approximately 20h later they were transferred into a silent room with standard light and temperature conditions, placed individually in transparent cages with paper pellets bedding for cat litter that effectively traps odor and moisture. In the morning (10 am and 2 pm, i.e. during the light period), animals assigned to SE were injected with a single dose of pilocarpine ip. (for details see below) and observed for at least 3 hours by experienced observers. Animals in the control group were injected with the same dose of saline. Animals that developed motor SE received after 1.5 hours of ongoing convulsive activity a single dose of paraldehyde ip (for details see below) and animals were put on a paper towel to prevent bedding aspiration. Approximately one hour later, the animals were injected subcutaneously with 0.9% NaCl (up to 3% of the body weight) to restore the volume loss. Animals without clear motor SE lasting for at least 1.5 hours were excluded from this experiment. During the recovery period that lasted approximately 5 days, animals were fed with a paste made of powdered rat chow and 1% glucose solution and they were forced to drink Waltham RC Rehydration support for small animals from a plastic pipette several times during the day. During the whole recovery period animals had free access to drinking bottles with fresh water and solid rat chow was placed on the bottom of the cage. The health status of animals and food and water consumption were monitored daily. At selected intervals, animals from the same experimental group (both control and experimental) were sacrificed by decapitation under deep anesthesia (ether). After the recovery period animals assigned for a three-month interval were transferred into the standard animal room for video-

monitoring and housed in standard cages in stable pairs for the whole study. The majority of animals assigned for a three-month interval experienced highly similar seizures, comprising of bilateral forelimb clonus with or without rearing and falling. Seizure frequency per day in individual animals is summarized in Supplementary figure 1. One animal with multiple generalized tonic-clinic seizures (N12 Supplementary figure 1) and one without seizures (N6 not included in the figure) during the monitoring period were excluded from analysis prior to MPS as potential outliers. One animal was euthanized during the monitoring due to the tumor.

For experiments with rat pups, dams with litters were transferred from breeding facilities at P9-10. At P11 animals were randomly assigned a code that allowed for their individual history to be followed throughout the entire study and animals were randomly divided into two groups (control and SE; n=5 each) and injected with LiCl (for details see below). For each time point, two litters composed of 5 controls and 5 SE animals were used. On the day of the experiment, pups were separated from their dams and transferred into a silent room with controlled conditions. The mean weight at P12 was 29.6±4.1g. Experiments were always performed in the same period of the day, between 10 am and 2 pm (i.e. during the light period). Animals were placed individually into small containers made of transparent plastics and they were maintained at +33±1ºC with a Physiological-Biological Temperature Controller (TMP-5b; Supertech; Hungary) to compensate for the immature thermoregulation at this age (Conklin and Heggeness, 1971) during the entire period of separation from their mothers. SE was induced with a single injection of pilocarpine (for details see below). After 1.5 hours of convulsive SE, animals were given a single dose of paraldehyde (for details see below), and approximately 30 min later they were injected subcutaneously with 0.5 ml saline to restore the volume loss. After the brief recovery, pups were returned to their dams (the duration of isolation from mothers in the control and SE groups was the same approximately 4 hours). The body weight of pups was checked daily and animals that did not gain any weight within 24 hours were given 0.5ml of saline subcutaneously to prevent dehydration.

SE induction – Animals in both age groups were injected intraperitoneally with LiCl (3 mmol/ml/kg; # L-0505, Sigma Chemical Co., St. Louis, MO) 24 hours prior to intraperitoneal injection pilocarpine (35 mg/ml/kg in P12 and 45 mg/ml/kg in adult animals; # P-6503, Sigma Chemical Co.) (Hirsch et al., 1992). The manifestation of the first clonic motor seizures was considered to be the beginning of SE. To decrease mortality, a single dose of paraldehyde (0.07 ml/kg for P12 rats and 0.6 ml/kg for adult animals; # 76260, Fluka Chemie AG, Buchs, Switzerland) was injected intraperitoneally 1.5 hours after the onset of SE. Control animals in both age groups were treated with equal doses of LiCl and paraldehyde, but the pilocarpine solution was replaced with saline.

The severity of motor SE was assessed using the following scoring system:

0 – normal behavior

1 – stereotypic behavior (face washing, scratching), isolated myoclonic jerks

2 –head bobbing, pivoting, swimming movements

3 – clonic seizures with preserved righting reflex

4 – repeated episodes of wild running

5 – generalized tonic clonic seizures with loss of righting reflex.

Animals were assigned a score for the most severe behavior observed. Latency to the onset of motor seizures was recorded. Mortality was recorded throughout the entire experimental period. Only rats that exhibited behavioral

manifestations of seizures progressing to forelimbs clonus (i.e., score 3) for at least 1 h and without periods of wild running and generalized tonic clonic seizures (score 4-5) were used for further studies. In acute (24 hours) and latent period (7 days after SE), spontaneous motor seizures (Racine 3-5) were occasionally observed during animal care in all P60 and more than 60% of P12 rats. Most P12 rats exhibited seizures in the first 3-4 days after SE. This observation is concordant with previous reports of seizures occurring during the so-called latent stage of epileptogenesis, which is typically considered as a seizure-free period lasting 1-6 weeks in rats with pilocarpine-induced TLE[1]. Hence, the term “latent stage” (in our case 7 days after SE) should be conceived as the early stage of TLE accompanied by pathological transformations with the possibility of occasional seizure occurrence, rather than truly seizure-free period[2].

Animals were sacrificed by decapitation under deep anesthesia 24 hours, 1 week and 3 months after SE, brains were quickly removed, both hippocampi were dissected and frozen in dry ice. Animals assigned to 3 months interval were video-monitored 24/7 for 1 week before scarifying with IP infrared Camera Edimax IC-3140W for wireless monitoring. Synology Surveillance Station 7 software was used for both registration and evaluation. Recordings were evaluated manually by an experienced observer. The incidence of motor seizures (Racine stage 3-5) was registered. EEG monitoring was not included in this study due to technical limitation rendering long term EEG impossible in rat pups (maternal care, skull growth, and ossification) and to prevent bias arising from the effect of anesthetics on the brain (especially during development) and inflammation induced by electrode implantation[3,4].

Table S1A – significantly dysregulated miRNAs in miR-Seq - adults - miRNAs identified by *DESeq2* or *limma* as significantly altered in animals after status epilepticus within each stage of epileptogenesis respectively. The list contains all miRNAs with fold-change above 1.4 and p-value <0.05 that reached the threshold of 500 reads in a given stage in adulthood epilepsy-onset group.

	acute							latent							chronic					
	DESeq2			limma2				DESeq2			limma2				DESeq2			limma2		
**miRNA**	**p-value**	**p-adj**	**FC**	**p-value**	**p-adj**	**FC**	**miRNA**	**p-value**	**p-adj**	**FC**	**p-value**	**p-adj**	**FC**	**miRNA**	**p-value**	**p-adj**	**FC**	**p-value**	**p-adj**	**FC**
let-7c-1-3p	-	-	-1,1	*	-	-1,4	miR-100-3p	-	+	1,4	**	-	1,4	let-7b-3p	**	*	1,6	**	+	1,5
let-7f-1-3p	*	+	1,5	*	-	1,4	miR-107-5p	**	+	-1,5	NA	NA	NA	miR-1193-3p	*	-	-1,5	-	-	-1,8
miR-124-5p	**	**	-1,5	**	*	-1,5	miR-1224	-	-	-1,4	*	-	-1,5	miR-125a-3p	-	-	-1,4	*	-	-1,4
miR-1247-5p	*	*	3,1	**	**	9,9	miR-132-3p	**	**	1,4	**	+	1,5	miR-129-2-3p	**	+	1,7	*	-	1,7
miR-129-2-3p	*	+	1,6	*	-	1,5	miR-135a-3p	**	**	1,5	**	-	1,4	miR-130b-3p	**	+	-3,5	NA	NA	NA
miR-132-3p	**	**	1,8	**	**	1,8	miR-135b-3p	*	-	-1,4	NA	NA	NA	miR-132-3p	*	-	1,4	*	-	1,4
miR-132-5p	**	**	1,6	**	**	1,6	miR-141-3p	*	*	1,7	NA	NA	NA	miR-132-5p	**	**	1,3	**	-	1,3
miR-134-3p	*	+	1,6	*	+	1,5	miR-142-3p	**	**	3,1	**	**	2,9	miR-133b-3p	*	-	-2,8	-	-	-1,2
miR-135a-3p	*	+	-1,4	**	-	-1,4	miR-142-5p	**	**	2,7	**	**	2,4	miR-135a-5p	**	*	1,6	**	*	1,6
miR-142-3p	**	**	1,9	**	**	1,8	miR-146a-5p	**	**	2,3	**	**	2,1	miR-138-5p	**	+	-1,4	-	-	-1,4
miR-142-5p	**	*	1,8	**	*	1,8	miR-154-5p	*	*	-1,4	-	-	-1,3	miR-142-3p	*	-	1,5	**	-	1,6
miR-144-3p	-	-	0,7	*	+	1,6	miR-155-5p	**	**	5,9	**	**	5,2	miR-142-5p	*	-	1,6	**	-	1,6
miR-152-3p	*	-	1,4	-	-	1,3	miR-15a-5p	-	-	1,3	*	-	1,4	miR-146a-5p	**	**	2,1	**	*	1,9
miR-153-3p	*	-	1,5	-	-	1,5	miR-15b-3p	**	*	1,8	**	+	1,8	miR-155-5p	**	**	2,9	**	+	2,5
miR-155-5p	**	**	6,5	**	**	7,1	miR-15b-5p	**	**	1,7	**	*	1,7	miR-17-5p	**	*	1,5	*	-	1,4
miR-17-5p	**	**	1,4	**	+	1,4	miR-17-1-3p	**	**	1,6	*	-	1,5	miR-187-3p	**	**	-2,0	**	*	-2,1
miR-181a-1-3p	*	*	-1,5	*	+	-1,5	miR-17-5p	**	**	1,9	**	**	2,0	miR-193a-5p	*	-	1,6	**	-	1,6
miR-181a-5p	**	**	-1,4	**	+	-1,4	miR-181a-2-3p	**	**	1,7	*	-	1,5	miR-203a-3p	**	*	1,6	**	+	1,6
miR-185-3p	-	-	-1,2	*	+	-1,4	miR-185-5p	*	+	-1,5	-	-	-1,2	miR-205	*	-	-2,2	-	-	-1,5
miR-18a-5p	*	-	1,4	-	-	1,3	miR-18a-5p	**	**	2,3	**	**	2,2	miR-206-3p	*	-	-2,1	*	-	-1,8
miR-193a-5p	-	-	-1,3	*	-	-1,8	miR-190b-5p	**	**	2,3	**	*	2,1	miR-20a-5p	*	-	1,4	*	-	1,4
miR-193b-3p	*	-	1,5	*	-	1,6	miR-19a-3p	**	**	2,2	**	**	2,2	miR-211-5p	NA	NA	NA	**	*	2,8
miR-19a-3p	*	-	1,5	-	-	1,3	miR-19a-5p	**	-	2,3	NA	NA	NA	miR-212-3p	**	**	1,7	**	+	1,6
miR-203a-3p	*	*	1,4	**	*	1,4	miR-19b-3p	**	**	1,5	**	*	1,6	miR-212-5p	**	+	1,5	*	-	1,5
miR-205	*	+	-1,7	*	-	-2,2	miR-20a-5p	**	**	1,9	**	**	1,9	miR-218a-5p	**	-	-1,5	*	-	-1,5
miR-212-3p	**	**	2,8	**	**	2,9	miR-211-5p	NA	NA	NA	**	*	2,2	miR-221-3p	**	**	1,7	-	-	-1,3
miR-212-5p	**	**	2,1	**	**	2,1	miR-212-3p	**	**	1,7	**	*	1,8	miR-223-3p	-	-	1,3	*	-	1,4
miR-21-5p	**	**	2,2	**	*	2,2	miR-212-5p	**	**	1,7	**	*	1,6	miR-23a-3p	-	-	1,3	**	-	1,5
miR-221-3p	**	**	-1,5	**	**	-1,5	miR-21-5p	**	**	2,1	**	*	2,0	miR-24-2-5p	*	-	1,5	**	-	1,5
miR-221-5p	**	*	-1,4	**	+	-1,5	miR-218a-2-3p	-	-	-1,6	*	-	-1,6	miR-27a-3p	-	-	1,3	**	+	1,5
miR-222-3p	**	*	-1,4	**	+	-1,4	miR-223-3p	*	*	1,6	*	-	1,4	miR-291b	*	-	8,9	NA	NA	NA
miR-223-3p	**	**	5,2	**	**	3,9	miR-23a-3p	**	**	1,5	**	*	1,5	miR-293-5p	**	-	7,3	NA	NA	NA
miR-27a-3p	*	+	1,3	**	*	1,5	miR-24-2-5p	**	**	1,4	*	-	1,4	miR-3120	*	-	3,4	NA	NA	NA
miR-298-5p	*	*	1,7	*	+	1,5	miR-293-5p	*	-	1,6	-	-	1,3	miR-339-3p	*	-	1,5	**	-	1,5
miR-29b-5p	*	*	-1,5	**	*	-1,5	miR-298-5p	**	*	1,4	**	+	1,5	miR-350	**	+	1,4	*	-	1,3
miR-31a-5p	*	+	1,6	-	-	1,4	miR-339-3p	**	-	1,7	**	*	1,6	miR-3547	*	-	-3,4	-	-	-1,1
miR-320-5p	-	-	0,5	**	**	27,2	miR-344b-1-3p	**	**	2,1	**	*	2,0	miR-3594-5p	*	-	-2,1	**	-	-2,6
miR-335	*	+	2,0	-	-	1,7	miR-344b-3p	**	**	1,7	**	-	1,7	miR-365-3p	**	+	1,6	**	-	1,5
miR-3473	*	*	3,5	**	**	9,2	miR-344b-5p	**	**	1,7	*	-	1,6	miR-381-5p	**	+	-1,7	*	-	-1,5
miR-34a-5p	*	-	-1,4	*	-	-1,4	miR-34b-3p	**	**	1,9	**	-	1,7	miR-449a-5p	*	-	-1,5	-	-	-1,5
miR-361-3p	**	**	-1,4	**	*	-1,4	miR-34c-3p	**	**	2,0	**	+	2,0	miR-483-5p	**	+	-7,3	NA	NA	NA
miR-365-3p	*	*	1,5	*	-	1,4	miR-34c-5p	**	**	1,9	*	-	1,6	miR-484	**	-	1,9	**	-	2,1
miR-376b-3p	**	**	1,6	**	*	1,5	miR-375-3p	*	*	2,3	-	-	1,3	miR-490-5p	*	NA	2,0	-	-	1,5
miR-377-3p	**	*	1,7	**	-	1,6	miR-501-3p	*	*	1,5	*	-	1,5	miR-496-5p	*	NA	-3,8	NA	NA	NA
miR-448-3p	*	+	2,7	-	-	2,0	miR-741-3p	*	-	3,3	NA	NA	NA	miR-504	*	-	-1,5	**	-	-1,6
miR-466c-5p	-	-	0,5	*	+	1,4	miR-7a-5p	**	**	-1,6	*	-	-1,5	miR-539-5p	*	-	-1,4	-	-	-1,3
miR-495	*	*	1,4	*	-	1,4	miR-7b	*	*	-1,5	-	-	-1,4	miR-676	*	-	-1,5	*	-	-1,4
miR-503-5p	*	-	-1,4	*	-	-1,4	miR-92a-3p	**	**	1,7	*	-	1,6	miR-764-3p	**	-	-2,6	-	-	-1,6
miR-504	**	**	-1,7	**	*	-1,8	miR-92b-5p	**	+	1,7	*	-	1,6	miR-7a-5p	**	*	-1,7	**	+	-1,9
miR-6215	-	-	0,5	**	-	-1,6	miR-98-3p	*	*	1,4	*	-	1,4	miR-7b	**	**	-1,9	**	+	-2,1
miR-665	*	*	1,4	**	+	1,4														
miR-667-3p	*	+	1,5	*	-	1,4														
miR-702-3p	*	-	1,5	*	-	1,5														
miR-873-5p	*	-	1,4	-	-	1,4														

** p < 0.01; * 0.01 < p < 0.05; - p > 0.05; + 0.05 < p-adj <0.1; NA not identified; FC fold change

Table S1B – **significantly dysregulated miRNAs in miR-Seq - infants** - miRNAs identified by *DESeq2* or *limma* as significantly altered in animals after status epilepticus within each stage of epileptogenesis respectively. List contains all miRNAs with fold-change above 1.4 and p-value <0.05 that reached the threshold of 500 reads in a given stage of infantile-onset epilepsy group.

acute						
	DESeq2			limma		
**miRNA**	**p-value**	**p-adj**	**FC**	**p-value**	**p-adj**	**FC**
miR-1247-5p	*	NA	-2,5	-	-	-1,4
miR-129-1-3p	*	-	1,5	-	-	2,8
miR-129-2-3p	*	*	1,7	**	+	1,6
miR-1298	*	+	-3,3	-	-	-1,4
miR-132-3p	**	**	1,8	**	**	1,9
miR-132-5p	**	**	2,2	**	**	2,2
miR-135b-5p	-	-	1,5	*	-	1,5
miR-139-5p	**	*	-1,5	**	*	-1,5
miR-142-3p	**	**	1,8	**	*	1,8
miR-142-5p	*	-	1,6	**	-	1,9
miR-155-5p	*	*	2,7	**	**	2,6
miR-182	*	-	1,7	*	-	1,8
miR-204-5p	*	+	-2,2	-	-	-1,3
miR-205	-	NA	-1,5	**	+	-1,7
miR-212-3p	**	**	2,9	**	**	2,8
miR-212-5p	**	**	2,8	**	**	3,0
miR-21-5p	**	**	3,0	**	**	3,1
miR-221-3p	*	+	-1,3	*	-	-3,6
miR-222-3p	*	*	-1,4	**	+	-1,4
miR-223-3p	*	-	1,7	**	+	1,7
miR-22-3p	*	-	1,4	*	-	1,6
miR-23a-3p	*	*	1,5	**	+	1,5
miR-24-2-5p	**	**	1,7	**	*	1,8
miR-24-3p	-	-	1,4	*	-	1,6
miR-27a-3p	**	**	1,5	**	*	1,7
miR-29b-3p	**	**	1,6	**	*	1,6
miR-3085	*	*	-1,6	-	-	-1,5
miR-330-3p	**	**	-1,5	**	*	-1,4
miR-3473	**	*	-1,9	-	-	-1,4
miR-34b-3p	*	-	-1,6	-	-	-1,3
miR-365-3p	*	+	1,5	**	+	1,5
miR-448-3p	*	+	-2,9	-	-	-1,5
miR-6215	*	NA	-2,7	*	-	-2,7
miR-7a-2-3p	*	*	-1,5	**	+	-1,5

latent						
	DESeq2			limma		
**miRNA**	**p-value**	**p-adj**	**FC**	**p-value**	**p-adj**	**FC**
miR-144-5p	**	+	-2,0	**	+	-2,0
miR-451-5p	*	-	-1,4	**	+	-1,5
miR-466b-4-3p	*	-	1,9	**	-	1,2
miR-99a-3p	*	-	-1,4	**	-	-1,4
miR-187-5p	*	-	2,4	-	-	1,2

chronic						
	DESeq2			limma		
**miRNA**	**p-value**	**p-adj**	**FC**	**p-value**	**p-adj**	**FC**
miR-128-2-5p	-	-	-1,3	*	-	-1,6
miR-1306-5p	-	-	-1,5	*	-	-1,5
miR-135a-5p	**	-	1,5	-	-	1,4
miR-137-3p	-	-	-1,3	*	-	-1,5
miR-140-5p	*	-	1,4	*	-	1,5
miR-148b-5p	**	**	2,2	**	-	2,2
miR-15a-5p	-	-	1,3	*	-	1,5
miR-187-5p	*	-	-3,1	-	-	-1,1
miR-204-5p	*	-	-1,7	-	-	-1,2
miR-22-3p	*	-	1,9	*	-	2,3
miR-24-2-5p	**	-	1,6	*	-	1,6
miR-24-3p	*	-	1,5	*	-	1,6
miR-301a-3p	*	-	-1,5	*	-	-1,7
miR-337-5p	*	-	1,3	*	-	1,5
miR-370-3p	*	-	1,6	*	-	1,8
miR-376a-3p	-	-	-1,4	*	-	-2,0
miR-431	**	+	2,2	-	-	1,5
miR-451-5p	*	-	-1,6	*	+	-3,4
miR-666-3p	*	-	1,7	*	-	1,8
miR-7a-1-3p	*	-	-1,4	-	-	-1,3
miR-99a-3p	-	-	-1,4	*	-	-1,8

** p < 0.01; * 0.01 < p < 0.05; - p > 0.05; + 0.05 < p-adj <0.1; NA not identified; FC fold change

Table S2 – **miRNAs selected for PCR validation –** list of miRNAs selected for miQPCR analysis based on results of sequencing (*DESeq2* and *limma*): 51 miRNAs in adulthood and 28 in infantile-onset TLE group. The list contains 60 unique miRNAs; both groups overlap in the case of 19 miRNAs.

Adult	P12
let-7b-3p	miR-129-2-3p
miR-124-5p	miR-132-3p
miR-1247-5p	miR-132-5p
miR-129-2-3p	miR-135a-5p
miR-132-3p	miR-139-5p
miR-132-5p	miR-142-3p
miR-134-3p	miR-142-5p
miR-135a-3p	miR-144-5p
miR-135a-5p	miR-148b-5p
miR-142-3p	miR-155-5p
miR-142-5p	miR-212-3p
miR-146a-5p	miR-212-5p
miR-155-5p	miR-21-5p
miR-15b-5p	miR-221-3p
miR-17-5p	miR-222-3p
miR-181a-1-3p	miR-223-3p
miR-181a-2-3p	miR-22-3p
miR-181a-5p	miR-23a-3p
miR-185-5p	miR-24-2-5p
miR-187-3p	miR-27a-3p
miR-18a-5p	miR-29b-3p
miR-190b-5p	miR-330-3p
miR-19a-3p	miR-3473
miR-19b-3p	miR-34b-3p
miR-203a-3p	miR-365-3p
miR-205	mir-451-5p
miR-20a-5p	miR-6215
miR-211-5p	miR-7a-2-3p
miR-212-3p	
miR-212-5p	
miR-21-5p	
miR-221-3p	
miR-221-5p	
miR-222-3p	
miR-223-3p	
miR-23a-3p	
miR-24-2-5p	
miR-27a-3p	
miR-298-5p	
miR-29b-5p	
miR-339-3p	
miR-344b-1-3p	
miR-3473	
miR-34b-3p	
miR-34c-3p	
miR-34c-5p	
miR-361-3p	
miR-365-3p	
miR-376b-3p	
miR-504	
miR-7b	

Table S3 – **predicted targets of miRNAs included in the validation group –** Brain physiology, function and development-related predicted targets of miRNAs identified with altered regulation by miRNA sequencing. miRNA targets were selected from a list produced by the Target Search tool (http://mirdb.org/cgi-bin/search.cgi) with a target score above 90 for all miRNAs included in the qPCR validation group. Validated targets of these miRNAs based on strong experimental evidence listed in miRTarbase (http://mirtarbase.cuhk.edu.cn) are underlined. miRNAs validated by the qPCR are displayed in bold font.

miRNA	gene	function	database
rno-let-7b-3p	*Clock*	clock circadian regulator	miRDB
	Gls	glutaminase	miRDB
	Net1	neuroepithelial cell transforming 1	miRDB
	Appbp2	amyloid beta precursor protein (cytoplasmic tail) binding protein 2	miRDB
	Bdnf	brain-derived neurotrophic factor	miRDB
	Camta1	calmodulin binding transcription activator 1	miRDB
	Dnali1	dynein, axonemal, light intermediate chain 1	miRDB
	Frs2	fibroblast growth factor receptor substrate 2	miRDB
	Loc100362819	autism susceptibility candidate 2-like	miRDB
	Nedd9	neural precursor cell expressed, developmentally down-regulated 9	miRDB
	Neurog2	neurogenin 2(axon guidance)	miRDB
	Npy1r	neuropeptide Y receptor Y1	miRDB
	Nyap2	neuronal tyrosine-phosphorylated phosphoinositide-3-kinase adaptor 2	miRDB
	Scn7a	sodium channel, voltage-gated, type VII, alpha	miRDB
	Spast	spastin (axon transport)	miRDB
	Syngr3	synaptogyrin 3 (synaptic vesicle protein)	miRDB
miR-124-5p	*Nxph1*	neurexophilin 1	miRDB
	Snx2	sorting nexin 2 (vesicular transport)	miRDB
miR-129-2-3p	*Gabra1*	gamma-aminobutyric acid (GABA) A receptor, alpha 1	miRDB
	Nptn	neuroplastin (synaptic membrane Ig)	miRDB
	Scn3b	sodium channel, voltage-gated, type III, beta	miRDB
miR-132-3p	*Arhgap32*	Rho GTPase activating protein 32	miRTarBase
	Foxo3	forkhead box O3	miRTarBase
	Grin2a	glutamate ionotropic receptor NMDA type subunit 2A	miRTarBase
	Grm3	glutamate metabothropic receptor 3	miRDB
	Kcna6	potassium voltage gated channel, shaker related subfamily, member 6	miRDB
	Lrrfip1	LRR binding FLII interacting protein 1	miRTarBase
	Pten	phosphatase and tensin homolog	miRTarBase
	Rasa1	RAS p21 protein activator 1	miRTarBase
	Slc26a4	solute carrier family 26 (anion exchanger), member 4	miRDB
	Slc6a1	solute carrier family 6 (neurotransmitter transporter), member 1	miRDB
miR-134-3p	*Syt1*	synaptotagmin I (exocytosis and vesicle trafficking in synaptic transport)	miRDB
miR-135a-5p	*Cacna1d*	calcium channel, voltage-dependent, L type, alpha 1D subunit	miRDB
	Cplx1	complexin 1 (binding SNARE, synaptic transport)	miRDB
	Slc5a7	solute carrier family 5 (sodium/choline cotransporter), member 7	miRDB
	Tnpo1	transportin 1	miRDB
miR-139-5p	*Elavl2*	ELAV like neuron-specific RNA binding protein 2	miRDB

	Morn4	MORN repeat containing 4 (neuroprotective)	miRDB
	Nptx1	neuronal pentraxin I (uptake of synaptic macromolecules)	miRDB
	Nxph1	neurexophilin 1	miRDB
	Pafah1b1	platelet-activating factor acetylhydrolase 1b, regulatory subunit 1	miRTarBase
miR-142-5p	*Bai3*	brain-specific angiogenesis inhibitor 3	miRDB
	Btg3	BTG anti-proliferation factor 3	miRTarBase
	Nedd1	neural precursor cell expressed, developmentally down-regulated 1	miRDB
	Slc12a1	solute carrier family 12 (sodium/potassium/chloride transporter), member 1	miRDB
miR-146a-5p	*Irak1*	interleukin-1 receptor-associated kinase 1	miRTarBase
	Nfkb1	nuclear factor kappa B subunit 1	miRTarBase
	Slc10a3	solute carrier family 10, member 3	miRDB
	Smad4	SMAD family member 4	miRTarBase
	Snap25	synaptosome associated protein 25	miRTarBase
	Traf6	TNF receptor associated factor 6	miRTarBase
miR-148b-5p	*Cacna1d*	calcium channel, voltage-dependent, L type, alpha 1D subunit	miRDB
	Clcc1	chloride channel CLIC-like 1	miRDB
miR-17-5p	*Ago1*	argonaute RISC catalytic component 1	miRDB
	Canx	calnexin	miRTarBase
	Caps2	calcyphosine 2 (calcium:sodium antiporter activity)	miRDB
	Golga2	golgin A2	miRTarBase
	Hspa4	heat shock protein family A (Hsp70) member 4	miRTarBase
	Hspa8	heat shock protein family A (Hsp70) member 8	miRTarBase
	Nedd4l	neural precursor cell expressed, developmentally down-regulated 4-like, E3 ubiquitin protein ligase	miRDB
	Npas2	neuronal PAS domain protein 2 (acquisition of specific types of memory)	miRDB
	Sept	septin 2 (polarized neurite outgrowth)	miRDB
	Slc24a2	solute carrier family 24 (sodium/potassium/calcium exchanger), member 2	miRDB
	Stx6	syntaxin 6 (vesicular transport)	miRDB
	Txnip	thioredoxin interacting protein	miRTarBase
	Wif1	Wnt inhibitory factor 1	miRTarBase
miR-181a-5p	*Bai3*	brain-specific angiogenesis inhibitor 3	miRDB
	Creb1	cAMP responsive element binding protein 1	miRTarBase
	Gabra1	gamma-aminobutyric acid (GABA) A receptor, alpha 1	miRDB
	Gls	glutaminase	miRDB
	Gpx1	glutathione peroxidase 1	miRTarBase
	Gria2	glutamate ionotropic receptor AMPA type subunit 2	miRTarBase
	Per2	period circadian clock 2	miRDB
	Slc7a11	solute carrier family 7 (anionic amino acid transporter light chain, xc- system), member 11	miRDB
	Tgm2	transglutaminase 2	miRTarBase
miR-185-5p	*Astn1*	astrotactin 1 (neuronal cell adhesion)	miRDB
	Bai1	brain-specific angiogenesis inhibitor 1	miRDB
	Ddn	dendrin (RNA pol II found in dentrites, modulated by sleep deprivation)	miRDB
	Neurod2	neuronal differentiation 2	miRDB
	Ntrk2	neurotrophic tyrosine kinase, receptor, type 2	miRDB
	Sgms1	sphingomyelin synthase 1	miRDB
	Slc26a9	solute carrier family 26 (anion exchanger), member 9	miRDB

	Slc8a1	solute carrier family 8 (sodium/calcium exchanger), member 1	miRDB
	Sv2b	synaptic vesicle glycoprotein 2b	miRDB
miR-190b-5p	*Bai3*	brain-specific angiogenesis inhibitor 3	miRDB
	Kcnq5	potassium voltage-gated channel, KQT-like subfamily, member 5	miRDB
	Neurod1	neuronal differentiation 1	miRDB
	Nlgn1	neuroligin 1 (ligand for neurexins; development and maturation of synaptic connections)	miRDB
miR-19a-3p	*Cntfr*	ciliary neurotrophic factor receptor	miRDB
	Kcna4	potassium voltage-gated channel, shaker-related subfamily, member 4	miRDB
	Npas2	neuronal PAS domain protein 2 (acquisition of specific types of memory)	miRDB
	Sema4c	sema domain, immunoglobulin domain (Ig), transmembrane domain (TM) and short cytoplasmic domain, (semaphorin) 4C	miRDB
	Slc24a3	solute carrier family 24 (sodium/potassium/calcium exchanger), member 3	miRDB
	Slc6a8	solute carrier family 6 (neurotransmitter transporter), member 8	miRDB
	Syt1	synaptotagmin I (exocytosis and vesicle trafficking in synaptic transport)	miRDB
miR-19b-3p	*Cntfr*	ciliary neurotrophic factor receptor	miRDB
	Grb2	growth factor receptor bound protein 2	miRTarBase
	Kcna4	potassium voltage-gated channel, shaker-related subfamily, member 4	miRDB
	Npas2	neuronal PAS domain protein 2 (acquisition of specific types of memory)	miRDB
	Sema4c	sema domain, immunoglobulin domain (Ig), transmembrane domain (TM) and short cytoplasmic domain, (semaphorin) 4C	miRDB
	Slc24a3	solute carrier family 24 (sodium/potassium/calcium exchanger), member 3	miRDB
	Slc6a8	solute carrier family 6 (neurotransmitter transporter), member 8	miRDB
	Syt1	synaptotagmin I (exocytosis and vesicle trafficking in synaptic transport)	miRDB
miR-203a-3p	*Cnr1*	cannabinoid receptor 1 (brain)	miRDB
	Gabarapl1	GABA(A) receptor-associated protein like 1	miRDB
	Gabra1	gamma-aminobutyric acid (GABA) A receptor, alpha 1	miRDB
	Kcnq5	potassium voltage-gated channel, KQT-like subfamily, member 5	miRDB
	Sema5a	sema domain, seven thrombospondin repeats (type 1 and type 1-like), transmembrane domain (TM) and short cytoplasmic domain, (semaphorin) 5A	miRDB
	Slc12a2	solute carrier family 12 (sodium/potassium/chloride transporter), member 2	miRDB
	Slc7a14	solute carrier family 7, member 14 amino-acid transport	miRDB
	Vegfa	vascular endothelial growth factor A	miRTarBase
miR-205	*Hiatl1*	hippocampus abundant transcript-like 1	miRDB
miR-20a-5p	*Atxn1l*	ataxin 1-like (brain development)	miRDB
	Clock	clock circadian regulator	miRDB
	Nedd4l	neural precursor cell expressed, developmentally down-regulated 4-like, E3 ubiquitin protein ligase	miRDB
	Npas2	neuronal PAS domain protein 2 (acquisition of specific types of memory)	miRDB
	Sept2	septin 2 (polarized neurite outgrowth)	miRDB
	Slc24a2	solute carrier family 24 (sodium/potassium/calcium exchanger), member 2	miRDB
	Stx6	syntaxin 6 (vesicular transport)	miRDB
	Sv2b	synaptic vesicle glycoprotein 2b	miRDB
miR-212-3p	*Foxo3*	forkhead box O3	miRTarBase
	Kcna6	potassium voltage gated channel, shaker related subfamily, member 6	miRDB
	Pten	phosphatase and tensin homolog	miRTarBase
	Rasa1	RAS p21 protein activator 1	miRTarBase
	Slc26a4	solute carrier family 26 (anion exchanger), member 4	miRDB
	Slc6a1	solute carrier family 6 (neurotransmitter transporter), member 1	miRDB

miR-212-5p	*Cbln2*	cerebellin 2 precursor (synaptogenesis induction)	miRDB
	Cnr1	cannabinoid receptor 1 (brain)	miRDB
	Slc6a8	solute carrier family 6 (neurotransmitter transporter), member 8	miRDB
miR-21-5p	*Ntf3*	neurotrophin 3	miRDB
	Pdcd4	programmed cell death 4	miRTarBase
	Peli1	pellino E3 ubiquitin protein ligase 1	miRTarBase
	Pten	phosphatase and tensin homolog	miRTarBase
	Tiam1	TIAM Rac1 associated GEF 1	miRTarBase
	Zbtb7a	zinc finger and BTB domain containing 7a	miRTarBase
miR-221-3p	*Bcl2l11*	BCL2 like 11	miRTarBase
	Cdkn1b	cyclin-dependent kinase inhibitor 1B	miRTarBase
	Cdkn1c	cyclin-dependent kinase inhibitor 1C	miRTarBase
	Ntf3	neurotrophin 3	miRDB
miR-221-5p	*Kcnq4*	potassium voltage-gated channel, KQT-like subfamily, member 4	miRDB
miR-222-3p	*Bcl2l11*	BCL2 like 11	miRTarBase
	Cdkn1b	cyclin-dependent kinase inhibitor 1B	miRTarBase
	Cdkn1c	cyclin-dependent kinase inhibitor 1C	miRTarBase
	Ntf3	neurotrophin 3	miRDB
miR-22-3p	*Grm5*	glutamate metabothropic receptor 5	miRDB
	Net1	neuroepithelial cell transforming 1	miRDB
	Trpm7	transient receptor potential cation channel, subfamily M, member 7	miRTarBase
miR-23a-3p	*Slc12a2*	solute carrier family 12 (sodium/potassium/chloride transporter), member 2	miRDB
	Stx12	syntaxin 12 (vesicle-mediated transport)	miRDB
miR-24-2-5p	*Kcnab1*	potassium voltage-gated channel, shaker-related subfamily, beta member 1	miRDB
miR-27a-3p	*Clcn3*	chloride channel, voltage-sensitive 3	miRDB
	Foxo3	forkhead box O3	miRTarBase
	Kcnk2	potassium channel, subfamily K, member 2	miRDB
	Nav2	neuron navigator 2	miRDB
	Nln	neurolysin (metallopeptidase M3 family)	miRDB
	Rxra	retinoid X receptor alpha	miRTarBase
	Slc35f1	solute carrier family 35, member F1	miRDB
	Slc36a4	solute carrier family 36 (proton/amino acid symporter), member 4	miRDB
	Slc6a1	solute carrier family 6 (neurotransmitter transporter), member 1	miRDB
	Slc7a11	solute carrier family 7 (anionic amino acid transporter light chain, xc- system), member 11	miRDB
	Thrb	thyroid hormone receptor beta	miRTarBase
miR-298-5p	*Cntn4*	contactin 4 (brain and neuron projection development; negative regulation of neuron differentiation)	miRDB
	Gabra1	gamma-aminobutyric acid (GABA) A receptor, alpha 1	miRDB
	Ncs1	neuronal calcium sensor 1	miRDB
	Snn	stannin (selective vulnerability of neuronal populations to the toxic effects of organotin compounds)	miRDB
miR-29b-3p	*Cav2*	caveolin 2	miRTarBase
	Cnr1	cannabinoid receptor 1 (brain)	miRDB
	Col12a1	collagen type XII alpha 1 chain	miRTarBase
	Col1a1	collagen type I alpha 1 chain	miRTarBase
	Col3a1	collagen type III alpha 1 chain	miRTarBase
	Col4a1	collagen type IV alpha 1 chain	miRTarBase

	Col5a1	collagen type V alpha 1 chain	miRTarBase
	Col5a2	collagen type V alpha 2 chain	miRTarBase
	Col5a3	collagen type V alpha 3 chain	miRTarBase
	Col7a1	collagen type VII alpha 1 chain	miRTarBase
	Dicer1	dicer 1, ribonuclease type III	miRDB
	Grip1	glutamate receptor interacting protein 1	miRDB
	Insig1	insulin induced gene 1	miRTarBase
	Itgb1	integrin subunit beta 1	miRTarBase
	Kctd20	potassium channel tetramerization domain containing 20	miRDB
	Mmp2	matrix metallopeptidase 2	miRTarBase
	Nav2	neuron navigator 2	miRDB
	Sypl2	synaptophysin-like 2 (neuroendocrine secretory granule membrane)	miRDB
	Vegfa	vascular endothelial growth factor A	miRTarBase
miR-29b-5p	*Net1*	neuroepithelial cell transforming 1	miRDB
	Nfasc	neurofascin (neurite outgrowth, neurite fasciculation, and organization of the axon initial segment)	miRDB
miR-330-3p	*Atp2b2*	ATPase, Ca++ transporting, plasma membrane 2	miRDB
	Cacnb4	calcium channel, voltage-dependent, beta 4 subunit	miRDB
	Efs	embryonal Fyn-associated substrate	miRDB
	Nfasc	neurofascin (neurite outgrowth, neurite fasciculation)	miRDB
	Nrg3	neuregulin 3	miRDB
	Slc18a2	solute carrier family 18 (vesicular monoamine transporter), member 2	miRDB
	Slc4a10	solute carrier family 4, sodium bicarbonate transporter, member 10(sodium-driven chloride bicarbonate exchange)	miRDB
	Snap23	synaptosomal-associated protein 23	miRDB
	Stx12	syntaxin 12 (vesicular transport)	miRDB
miR-344b-1-3p	*Olr59*	olfactory receptor 59	miRDB
miR-3473	*Dbx2*	developing brain homeobox 2	miRDB
	Slc6a8	solute carrier family 6 (neurotransmitter transporter), member 8	miRDB
miR-34b-3p	*Clock*	clock circadian regulator	miRDB
miR-34c-3p	*Clock*	clock circadian regulator	miRDB
miR-361-3p	*Nrgn*	neurogranin (neuron specific protein kinase C substrate)	miRDB
	Sez6	seizure related 6 homolog (mouse)	miRDB
	Sirt6	sirtuin 6	miRDB
	Slc25a22	solute carrier family 25 (mitochondrial carrier, glutamate), member 22	miRDB
	Slc38a3	solute carrier family 38, member 3 (glutamine)	miRDB
	Sorcs2	sortilin-related VPS10 domain containing receptor 2	miRDB
miR-365-3p	*Synj1*	synaptojanin 1	miRDB
miR-376b-3p	*Slc6a1*	solute carrier family 6 (neurotransmitter transporter), member 1	miRDB
	Dscaml1	Down syndrome cell adhesion molecule-like 1	miRDB
miR-7a-2-3p	*Olig2*	oligodendrocyte lineage transcription factor 2	miRDB
miR-7b	*Parp1*	poly (ADP-ribose) polymerase 1	miRTarBase
	Sept8	septin 8 (GTP binding (inferred) in myelin sheath)	miRDB
	Snca	synuclein, alpha (non A4 component of amyloid precursor; regulation of synaptic vesicle biogenesis)	miRDB
	Vdac1	voltage-dependent anion channel 1	miRDB

Table S4 – **Literature review** - overview of miRNAs identified with aberrant expression after SE. The list contains miRNAs identified by MPS and selected for PCR validation and their previous association with epilepsy in published studies. expr. – altered expression level; ref. – reference of detected altered expression level, Adults – adult epilepsy patients, Children – children with epilepsy

**Organism**	**Adult rat**		**Mouse**		**Adults**		**Rat pups**		**Children**	
**miRNA**	expr.	ref.	expr.	ref.	expr	ref.	expr.	ref.	expr.	ref.
let-7b-3p	-1	5								
miR-124-5p	-1	5,6	-1	7						
miR-129-2-3p	1	8	1	7	1	9,10				
miR-132-3p	1	5,6,8,11	1	7,12,13			1	14,15	1	14,15
miR-132-5p	1	6,8					1	14	1	14
miR-134-3p	1;-1	5,16	1	13,17						
miR-135a-5p	1	8	1	7	1	9				
miR-139-5p	-1;1	5,6,8,16	1	13						
miR-142-3p	1	5	1	7	1	10				
miR-142-5p	1	5	1	7	1	10				
miR-144-5p	1;-1	8,18			1	10,19				
miR-146a-5p	1	6,8,11,16,20	1	7	1	19,20	1	21	1	21
miR-155-5p	1	5,22					1	23	1	23
miR-17-5p	1	5	1	7	1	9				
miR-181a-1-			-1	7						
miR-181a-5p	-1	5	-1	7			1	24	1	24
miR-185-5p	-1	5,16			-1	25				
miR-187-3p	-1	6,8,26	-1	7	-1	12				
miR-205	-1	6								
miR-20a-5p	1	5			1	9				
miR-212-3p	1	6,8	1	7						
miR-212-5p	1	5,6,8								
miR-21-5p	1;-1	5,6,8,11	1	7,13			1	14	1	14
miR-221-3p	-1	5			-1	9	1	24	1	24
miR-221-5p					-1	19				
miR-222-3p	-1	5	1	7	-1	9	-1	24	-1	24
miR-223-3p	1	8	1	7	1	12				
miR-22-3p	1	5,11	1	7						
miR-23a-3p	1	6,8,16,18	1	7						
miR-24-2-5p	1	6,8	1	7						
miR-27a-3p	1	8,18	1;-1	7,13	1	9				
miR-298-5p	1	22	-1	7						
miR-29b-3p	-1;1	5,6,11	1	7						
miR-29b-5p	-1	6								
miR-330-3p	-1	6	-1	7,13	-1	12				
miR-339-3p			-1	7						
miR-34b-3p	1;-1	5,8	-1	7						
miR-34c-3p										
miR-34c-5p	1	6,8	1	27						
miR-361-3p					1	28				
miR-376b-3p	-1	5								
miR-451-5p	1	8	1	13	1	27				
miR-503-5p	1	5								
miR-7a-2-3p	-1	6								
miR-7b	-1	6								

-1 downregulated in epilepsy; 1 upregulated in epilepsy

Table S5 - List of validated miQPCR primers - List of miQPCR primers specific for miRNAs included in the validation group with confirmed standard curve efficiency between 90-110% and correlation coefficient (R^2) of 0.99 or higher. All listed primers produce a single melting peak in PCR reaction performed on cDNA reverse transcribed from rat total RNA. The melting temperatures (Tm) were calculated by freely available "Tm calculator" tool (Applied Biosystems) for all individual sequences.

miRNA	primer	Tm [°C]
rno-let-7b-3p	ACAACCTACTGCCTTCCCG	58.6
rno-miR-124-5p	TCACAGCGGACCTTGATG	59.3
rno-miR-1247-5p	CCGTTCGTCCCCGGAG	62.1
rno-miR-129-2-3p	CCCTTACCCCAAAAAGCATG	61.6
rno-miR-132-3p	ACAGTCTACAGCCATGGTCGG	60.93
rno-miR-132-5p	TGGCTTTCGATTGTTACTGG	58.8
rno-miR-134-3p	GGGCCACCTAGTCACCAAG	59.0
rno-miR-135a-5p	TATGGCTTTTTATTCCTATGTGAGG	58.5
rno-miR-139-5p	CAGTGCACGTGTCTCCAGG	61.6
rno-miR-142-3p	TGTAGTGTTTCCTACTTTATGGAGG	56.7
rno-miR-142-5p	CATAAAGTAGAAAGCACTACTGGC	55.4
rno-miR-146a-5p	GAGAACTGAATTCCATGGGTTG	60.7
rno-miR-148b-5p	AAGTTCTGTTATACACTCAGGGGC	60.0
rno-miR-155-5p	GCTAATTGTGATAGGGGTGGC	59.3
rno-miR-15b-5p	CGAATCATTATTTGCTGCTCTAGG	60.1
rno-miR-17-5p	AAAGTGCTTACAGTGCAGGTAGG	59.9
rno-miR-181a-5p	CAACGCTGTCGGTGAGTG	58.3
rno-miR-185-5p	AGAGAAAGGCAGTTCCTGAGG	60.0
rno-miR-18a-5p	TAAGGTGCATCTAGTGCAGATAGG	58.5
rno-miR-190b-5p	TGATATGTTTGATATTAGGTTGGC	55.9
rno-miR-19a-3p	GTGCAAATCTATGCAAAACTGAG	57.6
rno-miR-19b-3p	AAATCCATGCAAAACTGAGG	57.7
rno-miR-205	ATTCCACCGGAGTCTGTGG	59.3
rno-miR-20a-5p	AAAGTGCTTATAGTGCAGGTAGGG	58.8
rno-miR-212-3p	GTCTCCAGTCACGGCCAG	60.4
rno-miR-212-5p	TGGCTCTAGACTGCTTACTGGG	59.5
rno-miR-21-5p	GCTTATCAGACTGATGTTGAGGC	60.3
rno-miR-221-3p	CATTGTCTGCTGGGTTTCG	58.65
rno-miR-222-3p	GCTACATCTGGCTACTGGGTG	58.28
rno-miR-223-3p	GTCAGTTTGTCAAATACCCCG	59.3
rno-miR-22-3p	GCTGCCAGTTGAAGAACTGTG	61.0
rno-miR-23a-3p	ATTGCCAGGGATTTCCG	61.6
rno-miR-24-2-5p	TGCCTACTGAGCTGAAACAGTG	60.6
rno-miR-27a-3p	CAGTGGCTAAGTTCCGCG	60.6
rno-miR-298-5p	GAGGGCTGTTCTTCCCG	57.63
rno-miR-29b-3p	GCACCATTTGAAATCAGTGTTG	60.4
rno-miR-29b-5p	GGTTTCACATGGTGGCTTAGG	60.2
rno-miR-330-3p	CACAGGGCCTGCAGAGAG	60.7
rno-miR-339-3p	CCTCGACGACAGAGCCAG	59.0
rno-miR-344b-1-3p	TATAACCAAAGCCCGACTGTG	58.6
rno-miR-3473	GGGCTGGAGAGATGGCTAG	59.9
rno-miR-34b-3p	CACTAACTCCACTGCCATCG	57.7
rno-miR-34c-3p	ACTAACCACACAGCCAGGG	57.0
rno-miR-34c-5p	CAGTGTAGTTAGCTGATTGCGG	60.0
rno-miR-361-3p	CAGGTGTGATTCTGATTCGTG	59.1
rno-miR-365-3p	CCCCTAAAAATCCTTATGGC	56.2
rno-miR-376b-3p	CATAGAGGAACATCCACTTGGC	60.9
rno-miR-451-5p	AAACCGTTACCATTACTGAGTTGG	60.5
rno-miR-504	TGGTCTGCACTCTGTCGG	57.7
rno-miR-6215	GGTTGCAGAGCCAGGG	58.8
rno-miR-7a-2-3p	AAGTCCCAGTCTGCCACAG	58.8
rno-miR-7b	GGAAGACTTGTGATTTTGTTGTG	57.2

Figure S1 – motor seizure occurrence in the chronic stage of adult-onset TLE

The figure shows the number of motor seizures detected in individual animals with adult-onset TLE over 7 day period of video-monitoring 3 months after induction of status epilepticus (prior to brain tissue collection). Animals (n = 13) were allocated numbers 2 to 14 based on the camera channels used for their monitoring. One animal was euthanized due to the tumor and one animal did not have any motor seizures during the monitoring period (N10 and N6, not displayed). Animal N12 had repeated generalized tonic-clonic seizures (GTCS). Animals N6,10 and 12 were excluded from further analysis.

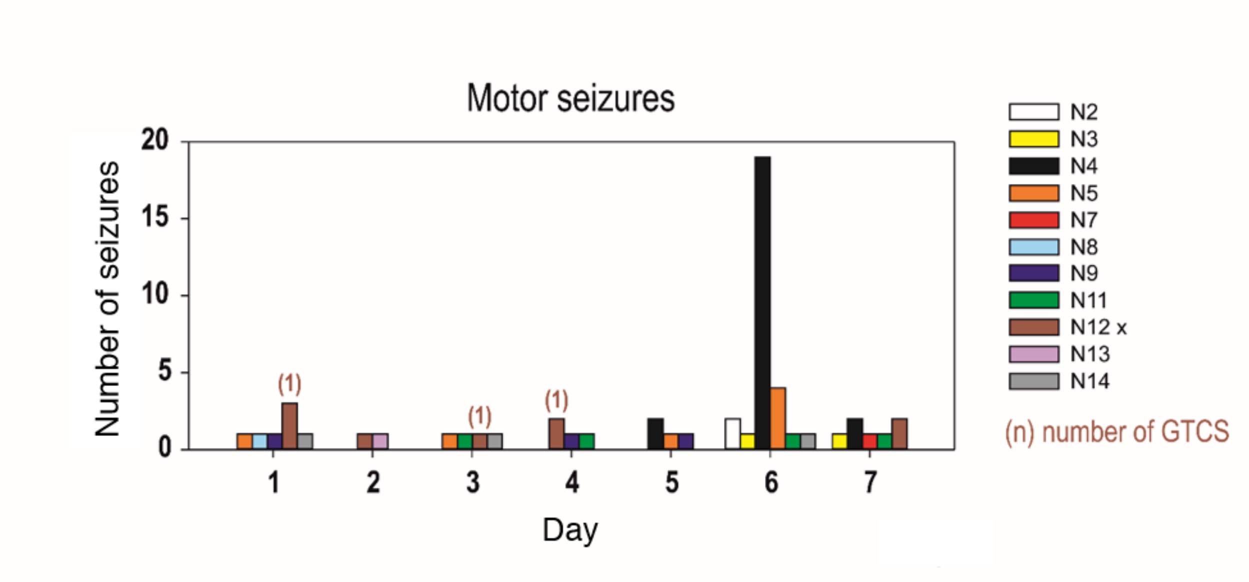

Figure S2 – **interactome maps of predicted targets** –brain physiology, function and development-related predicted targets of miRNAs identified with altered regulation by miRNA sequencing. Displayed genes linked with individual miRNAs were identified as putative targets by Target Search tool (http://mirdb.org/index.html) with a score over 90 or as validated brain-expressed targets listed in miRTarbase. Interactome maps display miRNA targets separately for each stage of epileptogenesis in adult- (**A**-acute; **B** –latent and **C**- chronic) and infantile- (**D**-acute; and **E**- chronic) onset TLE in rats. Line thickness represents miRNA expression fold change between TLE and control rats, while colours indicate up- (red) or downregulation (blue) in post-SE animals.

A- *Acute stage of adult-onset TLE*

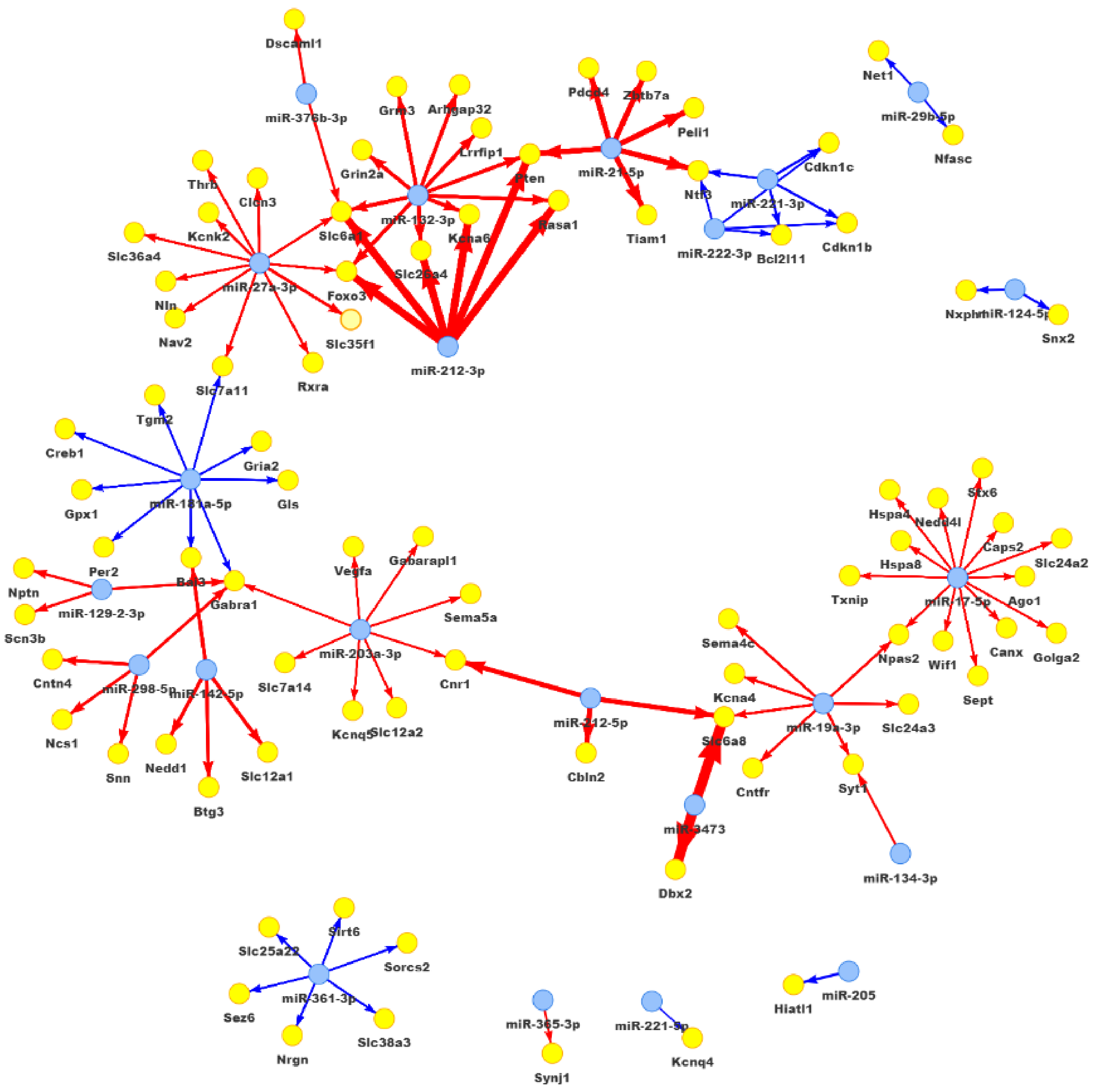

B- *Latent stage of adult-onset TLE*

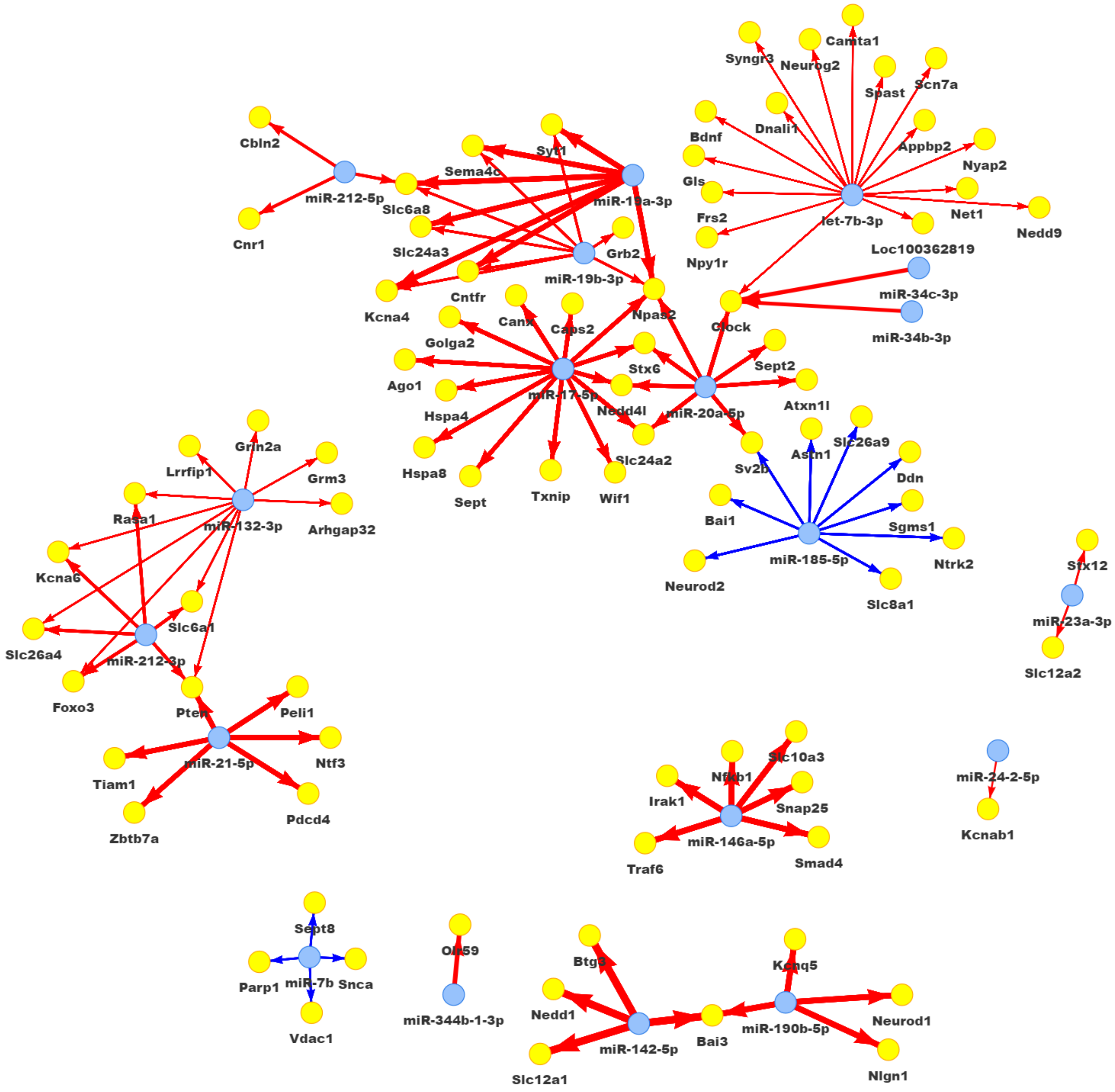

C- Chronic stage of adult-onset TLE

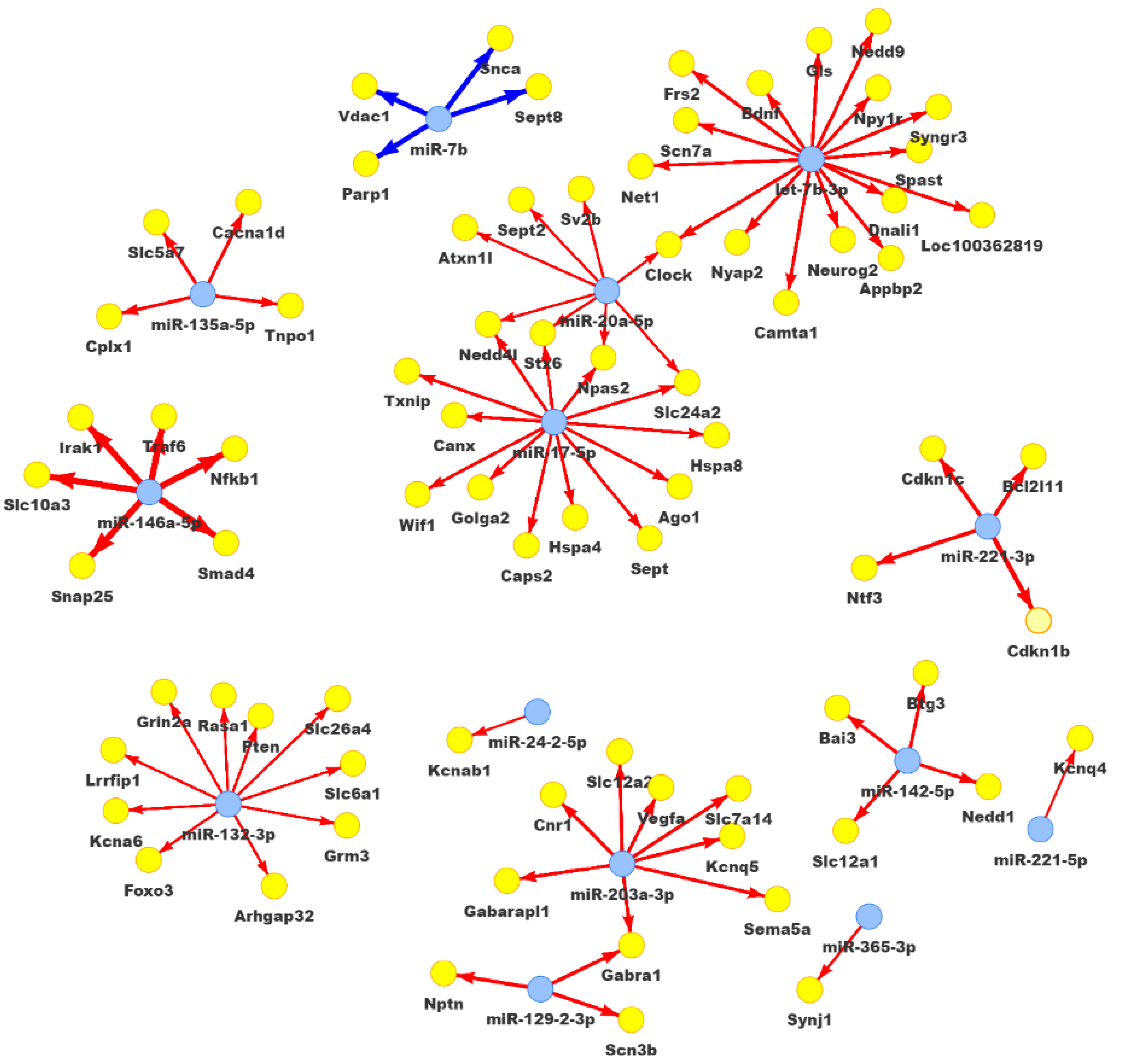

D- Acute stage of infantile-onset TLE

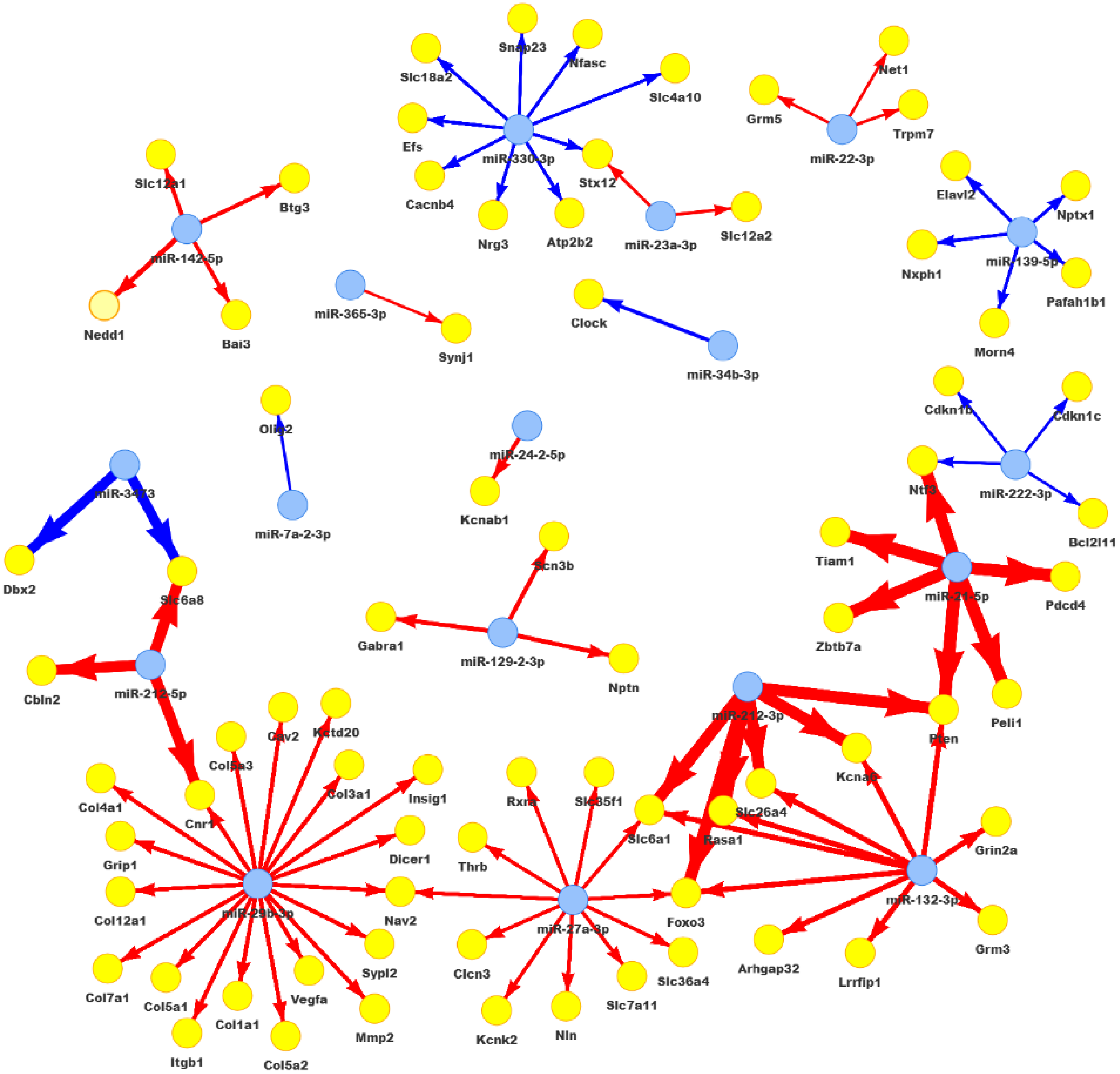

E- Chronic stage of infantile-onset

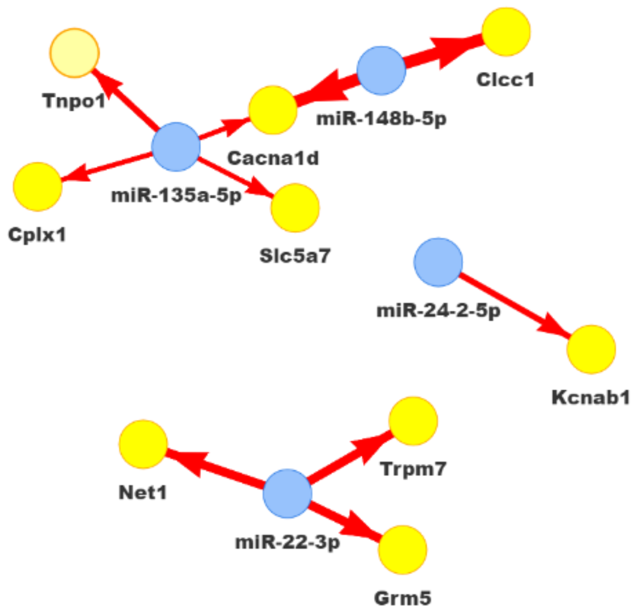

References

1. Löscher, W., Hirsch, L. J. & Schmidt, D. The enigma of the latent period in the development of symptomatic acquired epilepsy - Traditional view versus new concepts. *Epilepsy and Behavior* **52**, 78–92 (2015).

2. Curia, G., Longo, D., Biagini, G., Jones, R. S. G. G. & Avoli, M. The pilocarpine model of temporal lobe epilepsy. *J. Neurosci. Methods* **172**, 143–57 (2008).

3. Salatino, J. W., Ludwig, K. A., Kozai, T. D. Y. & Purcell, E. K. Glial responses to implanted electrodes in the brain. *Nat. Biomed. Eng.* **1**, 862–877 (2017).

4. ZHANG, F. *et al.* Emulsified isoflurane anesthesia decreases brain-derived neurotrophic factor expression and induces cognitive dysfunction in adult rats. *Exp. Ther. Med.* **8**, 471–477 (2014).

5. Risbud, R. M. & Porter, B. E. Changes in MicroRNA Expression in the Whole Hippocampus and Hippocampal Synaptoneurosome Fraction following Pilocarpine Induced Status Epilepticus. *PLoS One* **8**, e53464 (2013).

6. Bot, A. M., Dębski, K. J. & Lukasiuk, K. Alterations in miRNA Levels in the Dentate Gyrus in Epileptic Rats. *PLoS One* **8**, e76051 (2013).

7. Kretschmann, A. *et al.* Different MicroRNA Profiles in Chronic Epilepsy Versus Acute Seizure Mouse Models. *J. Mol. Neurosci.* **55**, 1–14 (2014).

8. Gorter, J. A. *et al.* Hippocampal subregion-specific microRNA expression during epileptogenesis in experimental temporal lobe epilepsy. *Neurobiol. Dis.* **62**, 508–520 (2014).

9. Kan, A. A. *et al.* Genome-wide microRNA profiling of human temporal lobe epilepsy identifies modulators of the immune response. *Cell. Mol. Life Sci.* **69**, 3127–3145 (2012).

10. Bencurova, P. *et al.* MicroRNA and mesial temporal lobe epilepsy with hippocampal sclerosis: Whole miRNome profiling of human hippocampus. *Epilepsia* **58**, 1782–1793 (2017).

11. Hu, K. *et al.* Expression profile of microRNAs in rat hippocampus following lithium-pilocarpine-induced status epilepticus. *Neurosci. Lett.* **488**, 252–7 (2011).

12. McKiernan, R. C. *et al.* Reduced mature microRNA levels in association with dicer loss in human temporal lobe epilepsy with hippocampal sclerosis. *PLoS One* **7**, e35921 (2012).

13. Jimenez-Mateos, E. M. *et al.* miRNA Expression Profile after Status Epilepticus and Hippocampal Neuroprotection by Targeting miR-132. *Am. J. Pathol.* **179**, 2519–2532 (2011).

14. Peng, J. *et al.* Expression Patterns of miR-124, miR-134, miR-132, and miR-21 in an Immature Rat Model and Children with Mesial Temporal Lobe Epilepsy. *J. Mol. Neurosci.* **50**, 291–297 (2013).

15. Ren, L., Zhu, R. & Li, X. Silencing miR-181a produces neuroprotection against hippocampus neuron cell apoptosis post-status epilepticus in a rat model and in children with temporal lobe epilepsy. (2016). doi:10.4238/gmr.15017798

16. Song, Y. *et al.* Temporal lobe epilepsy induces differential expression of hippocampal miRNAs including let-7e and miR-23a/b. *Brain Res.* **1387**, 134–140 (2011).

17. Jimenez-Mateos, E. M. *et al.* Silencing microRNA-134 produces neuroprotective and prolonged seizure-suppressive effects. *Nat. Med.* **18**, 1087–94 (2012).

18. Hu, K. *et al.* MicroRNA expression profile of the hippocampus in a rat model of temporal lobe epilepsy and miR-34a-targeted neuroprotection against hippocampal neurone cell apoptosis post-status epilepticus. *BMC Neurosci.* **13**, 115 (2012).

19. Wang, J. *et al.* Genome-wide circulating microRNA expression profiling indicates biomarkers for epilepsy. *Sci. Rep.* **5**, 9522 (2015).

20. Aronica, E. *et al.* Expression pattern of miR-146a, an inflammation-associated microRNA, in experimental and human temporal lobe epilepsy. *Eur. J. Neurosci.* **31**, 1100–1107 (2010).

21. Omran, A. *et al.* Interleukin-1β and microRNA-146a in an immature rat model and children with mesial temporal lobe epilepsy. *Epilepsia* **53**, 1215–1224 (2012).

22. Liu, D.-Z. *et al.* Brain and blood microRNA expression profiling of ischemic stroke, intracerebral hemorrhage, and kainate seizures. *J. Cereb. Blood Flow Metab.* **30**, 92–101 (2010).

23. Usman Ashhab, M. *et al.* Expressions of Tumor Necrosis Factor Alpha and MicroRNA-155 in Immature Rat Model of Status Epilepticus and Children with Mesial Temporal Lobe Epilepsy. *J. Mol. Neurosci.* **51**, 950–958 (2013).

24. Usman Ashhab, M. *et al.* microRNA s (9, 138, 181A, 221, and 222) and mesial temporal lobe epilepsy in developing brains. *Transl. Neurosci.* **4**, 357–362 (2013).

25. Wang, X. *et al.* Serum MicroRNA-4521 is a Potential Biomarker for Focal Cortical Dysplasia with Refractory Epilepsy. *Neurochem. Res.* **41**, 905–912 (2016).

26. Alsharafi, W. A., Xiao, B., Abuhamed, M. M., Bi, F.-F. & Luo, Z.-H. Correlation Between IL-10 and microRNA-187 Expression in Epileptic Rat Hippocampus and Patients with Temporal Lobe Epilepsy. *Front. Cell. Neurosci.* **9**, 466 (2015).

27. McKiernan, R. C. *et al.* Expression profiling the microRNA response to epileptic preconditioning identifies miR184 as a modulator of seizure-induced neuronal death. *Exp. Neurol.* **237**, 346–354 (2012).

28. Kaalund, S. S. *et al.* Aberrant expression of miR-218 and miR-204 in human mesial temporal lobe epilepsy and hippocampal sclerosis-Convergence on axonal guidance. *Epilepsia* **55**, 2017–2027 (2014).
